# Diet Quality Indices Used in Australian and New Zealand Adults: A Systematic Review and Critical Appraisal

**DOI:** 10.3390/nu12123777

**Published:** 2020-12-09

**Authors:** Hlaing Hlaing-Hlaing, Kristine Pezdirc, Meredith Tavener, Erica L. James, Alexis Hure

**Affiliations:** 1 School of Medicine and Public Health, University of Newcastle, Callaghan, Newcastle, NSW 2308, Australia; kristine.pezdirc@newcastle.edu.au (K.P.); meredith.tavener@newcastle.edu.au (M.T.); erica.james@newcastle.edu.au (E.L.J.); alexis.hure@newcastle.edu.au (A.H.); 2 Hunter Medical Research Institute, New Lambton Heights, Newcastle, NSW 2305, Australia

**Keywords:** adults, dietary assessment, diet quality, evaluation, index, systematic review, validity

## Abstract

Distilling the complexity of overall diet into a simple measure or summative score by data reduction methods has become a common practice in nutritional epidemiology. Recent reviews on diet quality indices (DQI) have highlighted the importance of sound construction criteria and validation. The aim of this current review was to identify and critically appraise all DQI used within Australian and New Zealand adult populations. Twenty-five existing DQI were identified by electronic searching in Medline and hand searching of reference lists. DQI were constructed based on the respective national dietary guidelines and condition-specific recommendations. For preferable features of DQI, six captured the dimensions of adequacy, moderation and balance; five had a nested structure; 12 consisted of foods, food groups and nutrients; 11 used metric scoring systems and most of those with metric scales used normative cutoff points. Food frequency questionnaires, either alone or with other methods, were the most common dietary assessment method used in 20 DQI. For evaluation of DQI, construct validity and relative validity are reported. Based on our critical appraisal, Dietary Guideline Index (DGI), Dietary Guideline Index-2013 (DGI-2013), Total Diet Score (TDS), Healthy Eating Index for Australian Adults-2013 (HEIFA-2013), and Aussie-Diet Quality Index (Aussie-DQI) were the preferred DQI used in Australian adults according to dimension, indicator selection, scoring criteria and evaluation. Further work is needed to enhance the construction of all Australian and New Zealand DQI, especially in terms of dimension and structure, for alignment with recommended construction criteria.

## 1. Introduction

In public health and epidemiology, there is often a need to condense the complex nature of dietary patterns and intake into a simple measure or summative score [1,2,3]. This is typically done in one of two ways. Data reduction techniques, like principal component, factor or cluster analysis, can be used to derive dietary patterns from comprehensive dietary data through multivariate analysis [4,5]. Diet quality indices (DQI) are the alternative, which apply a priori scoring criteria for adherence to dietary recommendations, like dietary guidelines [4,6,7]. Diet quality (DQ) can then be used to quantify chronic disease risk and mortality across population [3]. In the present review we focus on predefined DQI that aims to summarize the overall diet into a single measure.

To evaluate people’s adherence to accepted guidelines and optimal eating behavior, DQI are developed [3]. The earliest DQI were constructed with reference to the American Dietary Guidelines [8,9,10] and the Mediterranean diet [11]. Other indices have been derived in accordance with country-specific dietary guidelines or modified based on previously developed indices.

Several reviews on DQI have been published [1,2,3,6,7,12]. The review by Waijer et al. [1] emphasized methodological issues of DQI for their compositions, scoring and validity. Another review [3] highlighted the importance of validation studies by means of biomarkers or intermediate-risk factors for potential applicability in both clinical and public health settings. Two recent reviews have focused on relevant index construction criteria [13,14]. Burggraf et al. discussed the contribution of a theoretical basis, including all dimensions of diet quality (adequacy, moderation, variety and balance) and current diet-health relationship knowledge in DQI construction [14]. A further recent systematic review conducted by Trijsburg et al. identified those DQI developed for both low- and middle-income countries (LMIC) and high-income countries [13]. The authors concluded that there is a need for sound metrics to assess diet quality and suggested the guidelines for DQI construction both in LMIC and high-income countries [13].

Both Burggraf et al. and Trijsburg et al. have reviewed some, but not all, of the DQI [8,11,15,16,17,18,19,20,21,22,23] that have been used in Australian and New Zealand adults, especially with consideration given to the construction methodology [14], age group and context [13]. However, these Australian and New Zealand tools are in among many other international tools, derived from slightly different international dietary guidelines on which they are based. Therefore, this review aims to systematically identify and critically appraise all DQI used specifically with Australian and New Zealand adults. The construction criteria developed by Burggraf et al. and supplemented by Trijsburg et al. [13,14] was used to critically appraise each DQI and identify those that performed best. Diet-disease associations were considered only for validity assessment.

## 2. Materials and Methods

The review process was conducted by applying methods recommended by the Center for Reviews and Dissemination (CRD), University of York [24] and the Preferred Reporting Items for Systematic Reviews and Meta-Analyses (PRISMA) statement [25]. The review protocol was registered with the PROSPERO, registration number CRD42020149720.

### 2.1. Developing the Search Strategy and Databases to Be Included

The electronic database search was conducted in Medline, Embase and Cumulative Index to Nursing and Allied Health Literature (CINAHL) for papers published between 2000 to 2019. The keywords used in the review were “diet*, healthy eating, food, nutri*” in combination with AND for “index, score, tool, indic*”, then combined with “AND” for “Australia or New Zealand” either in the title, abstract, subject headings or original title. Filters were used to limit the results to those conducted in the human and English language. In addition, the reference lists of the retrieved papers were hand-searched to identify the relevant studies that were not detected by the electronic search strategy.

### 2.2. Inclusion/Exclusion Criteria for Eligible Studies

Studies were included if (1) participants were human adults aged 18 years and over, (2) participants were based in Australia and/or New Zealand, (3) DQ was assessed by using indices based on latest or current nutritional guidelines or recommendations, (4) scores, indices or tools that summarize the overall diet into a single value, (5) study designs were observational or experimental, (6) they described the development, application and/or validity of the DQ measure by means of nutrient adequacy, biomarkers or health outcomes.

Studies were excluded if (1) conducted in animals, (2) conducted among children, adolescents, pregnant and/or lactating women, (3) used a posteriori or empirically derived dietary pattern by using factor analysis (principal component analysis) or cluster analysis, (4) scores, indices or tools focused on one or more nutrients (e.g., fat), but not the overall diet, (5) the study design was a review, commentary, editorial, conference proceeding or theses, and (6) non-English publications.

### 2.3. Screening Procedure

**Figure 1:**
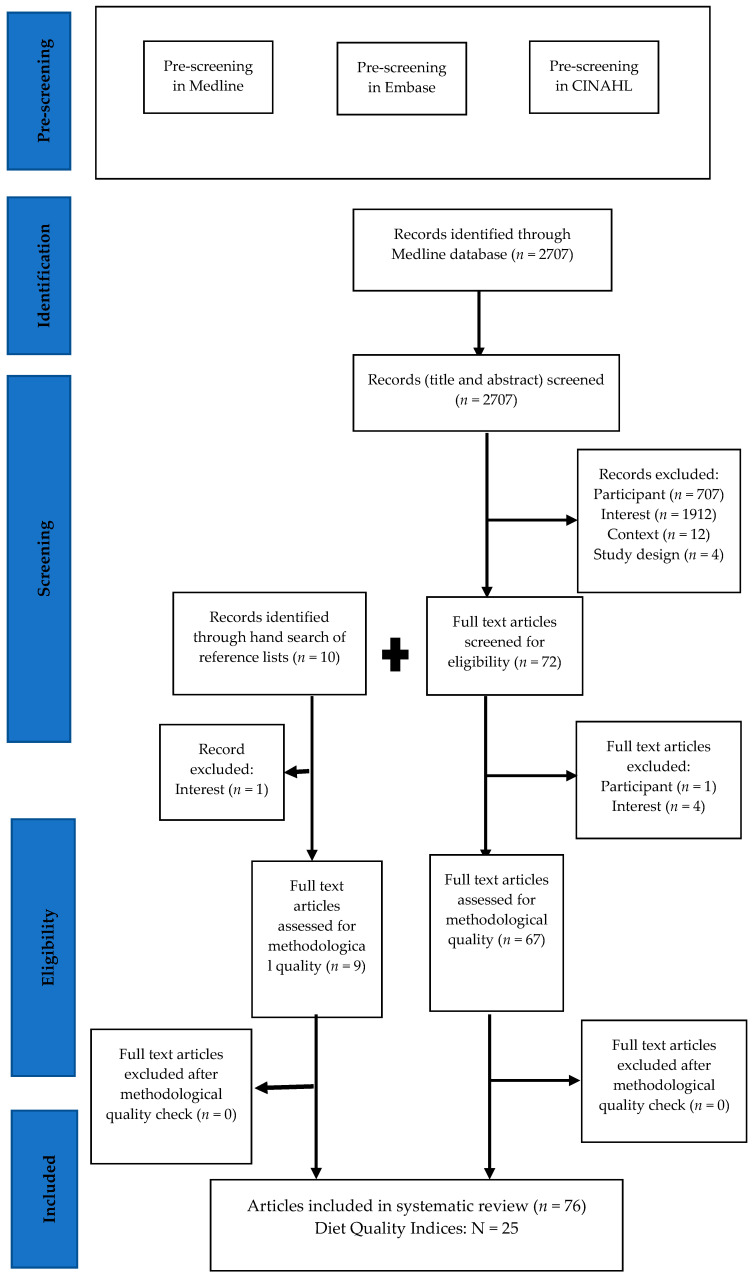
Flow chart for the systematic review process.

Titles of all articles in Medline, Embase and CINAHL were first screened by a single reviewer (HHH). After hand searching the references from Medline, Embase and CINAHL, only one additional paper was found. Therefore, duplicate screening was restricted to the Medline database only (See in Figure 1). Eligibility was assessed by two independent reviewers (HHH, KP) based on information contained in the title, abstract and description/MESH headings. If eligibility was unclear, the paper was referred to a third reviewer (AH) and discussed to reach a consensus.

Based on the information contained in the title and abstract, relevance to the review was assessed first. Articles were excluded by using a hierarchical approach based on population, the topic of interest, context and study design. The full texts were retrieved and screened to check for compliance with eligibility. Some were excluded according to the inclusion and exclusion criteria. The reference lists of the articles were also examined for possible inclusion in the review. Then the second round of screening was conducted by the first two reviewers, and the eligibility of the publication was considered. Only the studies deemed eligible were data extracted.

### 2.4. Quality of the Evidence

The quality of each article was assessed by two independent reviewers (HHH and KP) using the American Dietetic Association Quality Criteria Checklist for primary research [26]. The Checklist addressed issues of relevance to practice and validity for scientific soundness. For each point on the Checklist, each reviewer assigned ‘Yes’ if the criterion was met, ‘No’ if the criterion was not met, ‘Unclear’ if the criterion was not clearly described, or ‘N/A’ if the criterion did not apply to the study. The answers were tabulated, and each study was rated as “positive”, “negative,” or “neutral”. Those articles assigned “yes” to six or more of the validity questions were considered methodologically rigorous and rated as “positive”. If the article was assigned “no” or “unclear” on at least one of the validity questions, it was rated as “neutral”. Articles assigned “no” or “unclear” on six or more of the validity questions were considered not meeting methodological quality and rated as “negative” (See in Table S1). Any discrepancy between the reviewers was resolved through discussion.

### 2.5. Data Extraction

Two independent reviewers (HHH, KP) extracted relevant data using a data extraction form designed specifically for this review, adapted from the Cochrane Review Group [27]. Critical appraisal of the included DQI was conducted by using construction criteria developed by Burggraf et al. and supplemented by Trijsburg et al. [13,14]. The features of DQI assessed in our study were based on theoretical framework; dimensions (adequacy, moderation, balance, variety); dimensional structure (unordered, ordered, nested); indicator selection (database and component types- food groups, nutrients or both); normalization methods which contain scaling procedure (dichotomous, ordinal, metric) and cutoff values (normative, percentile, uniform, group-specific); and aggregation technique (unweighted, weighted) [14], the inclusion of healthy or unhealthy components, and evaluation of DQI [13]. Preferable features of DQI were the inclusion of adequacy, moderation and balance in dimension; nested structure; both food groups and nutrients in indicator selection; both healthy and unhealthy components; metric scaling procedure; normative cutoff points; weighted aggregation technique; and evaluation of DQI. Disagreement between reviewers (HHH, KP) was resolved by discussion and/or assessment by a third reviewer (AH).

The data extracted include information about the overview of the most current DQI (ordered chronologically) (Table 1); overview of components of DQI (Table S2); summary findings of studies investigating DQI, health outcomes and non-health-related factors (Table 2) and critical appraisal of DQI by previously suggested recommendations (Table 3).

## 3. Results

### 3.1. Australian and New Zealand Diet Quality Indices

In total, 76 relevant articles describing 25 DQI were identified. Of the 25 indices identified, 24 had been applied in Australia [8,11,15,16,17,18,19,21,22,23,28,29,30,31,32,33,34,35,36,37,38,39,40,41] and one in New Zealand [20]. Thirty-six percent (*n* = 9/25) were modified versions of an original tool, leaving 16 different indices, as summarized in Table 1.

Nearly half of the DQI (*n* = 12) aimed to operationalize the Australian Dietary Guidelines [16,17,18,19,28,29,30,33,36,37,38,42] and one operationalized New Zealand’s Food and Nutrition Guidelines for Healthy Adults [43]. A further three indices [8,31,41] were based on US dietary guidelines [44,45,46]. Nine of the identified indices operationalized condition-specific recommendations for a Mediterranean diet [11,21,39,47], chronic diseases [48,49,50,51,52], lowering blood pressure [53], cardiovascular disease prevention [54] and anti-inflammation [34]. Among recent Australian DQI (*n* = 8), 50% of their original indices [19,28,29,33] had been modelled or adapted from US indices [9,10,41,55,56,57,58].

The included DQI has been used across a variety of adults, based on sex and age. Of all of the DQI, five were validated and/or applied with women only [15,29,31,37,41] and 20 of them with both men and women [8,11,16,17,18,19,20,21,22,23,28,30,32,33,35,36,38,39,40,47]. Furthermore, the DQI was validated and/or applied across a broad age range of adults aged 18 and above (*n* = 12), or more specifically, middle-aged adults (*n* = 5), older adults (*n* = 4), middle-aged and older adults (*n* = 2), young university adults (*n* = 1) or a mean age was reported (*n* = 2).

**Table 1:** Overview of the most current diet quality indices used in Australian and New Zealand adults.

Index	Reference	Theoretical or Epidemiological Basis	Original Tool (Local or International)	Modified or Adapted Intermediate Tool	Components	Evaluation of Diet Quality Index
**Based on Australian Dietary Guidelines**						
Australian Healthy Eating Index (Aust-HEI)	Australian Institute of Health and Welfare, 2007 [28]	Australian Guide to Healthy Eating (AGHE), 1998 [59], Dietary Guidelines for Australian Adults, 2003 [60]	-	Variety score from previous food variety score [56] and Diet Quality Index-Revised [57]; Healthy choice score from Recommended Food Score [41]	Variety; healthy choices; fruit; vegetable; low-fat milk; trim fat meat; high saturated fat, low nutrient density food	Nutrient intakes, demographic and lifestyle characteristics, general health status
Australian Recommended Food Score-1 (ARFS-1)	Collins et al. 2015 [42]	Australian Dietary Guidelines-2013 [61]	Recommended Food Score [55]	Australian Recommended Food Score [29], Australian Child and Adolescent Recommended Food Score [62]	Vegetable; fruit; protein foods; grains; dairy; fats; alcohol	Nutrient intakes
Commonwealth Scientific and Industrial Research Organization- Healthy Diet Score (CSIRO HDS)	Hendrie et al. 2017 [63]	Australian Dietary Guidelines-2013 [61]	-	Dietary Guideline Index [19]	Variety; vegetables; fruits; whole-grain cereals; meat and alternatives; dairy and alternatives; water; discretionary foods; trim fat; fats and oils; salt; sugar; alcohol	Mean dietary score component
Total Diet Score (TDS)	Russell et al. 2013 [33]	Australian Guide to Healthy Eating (AGHE), 1998 [59], Dietary Guidelines for Australian Adults, 2003 [60]	US 2005 Dietary Guidelines Adherence Index [58]	Australian Healthy Eating Index [28]	Vegetables, legumes and fruit; cereals/whole grains; lean meats and alternatives; dairy and alternatives; saturated fat; sodium; alcohol; sugar; extra food; physical activity	All-cause mortality
Aussie-Diet Quality Index (Aussie-DQI)	Zarrin et al. 2013 [18]	Australian Guide to Healthy Eating (AGHE), 1998 [59], Dietary Guidelines for Australian Adults, 2003 [60]	Australia National Health Priority Area (ANHPA) [64]	Australian Healthy Eating Index [28], Dietary Guideline Index [19]	Vegetables; fruits; dairy products; meat and alternatives; cereals; saturated fat; sugar; alcohol; processed meat; salt/sodium; variety	Sociodemographic and lifestyle characteristics, cancer mortality
Healthy Eating Index for Australian-2013 (HEIFA-2013)	Roy et al. 2016 [16]	Australian Dietary Guidelines-2013 [61]	-	-	Discretionary foods; vegetables; fruits; whole grains; protein foods; dairy; water; saturated fat; sodium; sugar; alcohol	Nutrient intakes
Australian Diet Quality Score (ADQS)	Froud et al. 2019 [30]	Recommended Daily Intake (RDI) of the Australian Dietary Guidelines (not specified)	-	-	Vegetable; fruits; whole grains; processed grains; dairy; proteins; nuts; seafood; fats ratio; extras ratio	Nil
**Based on New Zealand Dietary Guidelines**						
Healthy Dietary Habits Index (HDHI)	Wong et al. 2017 [20]	New Zealand food and nutrition guidelines for healthy adults [43]	Healthy Dietary Habit Score for New Zealand adolescents [65]	-	Red meat; chicken; fish/shellfish; milk; spread; low-fat foods; fries; bread; fruits; vegetable; soft drinks; breakfast; fast foods; added salt; low salt food	Nutrient intakes, biomarker
**Based on US Dietary Guidelines**						
Diet Quality Index-Revised (DQI-R)	Haines et al. 1999 [8]	1989-Dietary recommendations from the US National Academy of Sciences and Dietary Guidelines for Americans [44], dietary reference intakes [45]	Diet Quality Index [10]	-	Total fat; saturated fat; cholesterol; fruits; vegetables; grains; calcium; iron; diversity; moderation	Nutrient intakes
Recommended Food Score (RFS)	Kant and Graubaud, 2000 [41]	1989-Dietary recommendations from the US National Academy of Sciences and Dietary Guidelines for Americans [44], epidemiological evidence [46]	Developed by Kant and Graubaud [41]	-	Fruits; vegetables; whole grains; lean meat or alternatives; low-fat dairy	Mortality
Not Recommended Food Score (NRFS)	Michels et al. 2002 [31]	1989-Dietary recommendations from the US National Academy of Sciences and Dietary Guidelines for Americans [44], epidemiological evidence [46]	US Dietary guidelines and results of large epidemiological studies [31]	-	Meat and its products; fried food; foods high in fat; others	Mortality
**Specific Dietary Pattern Recommendations**						
Mediterranean Diet Scale (MDS)	Trichopoulou et al. 2005 [39]		Assessment of adherence to a Mediterranean diet developed by Trichopoulou et al. [21]	-	Grains; vegetables; nuts and legumes; fruits; fish; olive oil; dairy products; red and processed meat; alcohol	Mortality
Dietary Approach to Stop Hypertension (DASH)	Fung et al. 2008 [15]	Guideline for lowering blood pressure [53]		-	Fruits; vegetables; nuts and legumes; whole grains; low-fat dairy; sodium; red and processed meat; sweetened beverage	CHD and stroke risk
Alternative Healthy Eating Index-2010 (AHEI-2010)	Chiuve et al. 2012 [22]	Foods and nutrients that lowered chronic diseases based on the Mediterranean diet [48,49,50,51,52]	Healthy Eating Index [9]	Alternative Healthy Eating Index (AHEI) [23]	Vegetables; fruits; nuts and soy protein; ratio of white to red meat; cereal fiber; trans-fat; ratio of polyunsaturated to saturated fatty acids; alcohol; multivitamin use	Chronic disease risk
Diet Quality Tool (DQT)	O’Reilly et al. 2012 [32]	Heart Foundation’s secondary prevention nutrition guidelines [54]	-	-	Vegetable; fruits; rice, pasta or noodle; breakfast cereals; bread; spread; milk; trim fat meat; takeaway meals; discretionary foods; fish; salt use	Nutrient intakes
Dietary Inflammatory Index (DII)	Shivappa et al. 2014 [66]	Literature-derived, population-based dietary inflammatory index [34]	Original DII [67]	-	Nutrients, spices, whole food and other	High-sensitivity C-reactive protein

### 3.2. Composition of Diet Quality Indices

The composition of the Australian and New Zealand DQI in this review included food groups, individual foods and beverages, nutrients, variety, and/or occasionally other lifestyle behaviors, such as physical activity and supplement use (Table S2). Thirteen indices were comprised exclusively of food and/or food groups [17,19,20,28,29,31,32,36,37,41,42,47,63] and twelve indices consisted of foods, food groups and nutrients [8,11,15,16,18,21,22,23,30,34,39,68].

Almost all indices (*n* = 23) included vegetable intake, ranging from 22 vegetable items represented by ARFS and 7 captured by RFS. Twenty-two indices included fruits, with 14 fruit items represented by ARFS and 7 by RFS. Whole grains or whole grain cereals were included in 14 indices [15,16,17,19,20,22,29,30,32,33,36,38,41,42], but units of measurement were different. For source of protein foods, meat and/or its alternatives were included in 23 out of 25 DQI (92%); of which lean meats was counted in five DQI [16,17,19,33,41], and gave points as proportion [17,19] or servings per day [16] or per week [33]. Twelve of the DQI included fish in their scoring [11,20,21,29,32,33,36,38,39,41,42,47], and nearly half of them (*n* = 5) gave points [11,21,33,39,47]. Majority of the indices (*n* = 20, 80%), except NRFS, DQI-R, AHEI, AHEI-2010 and DII, comprised of dairy products, in which 30% of them (6/20) recorded low-fat dairy [15,16,41] or low-fat/skimmed milk [28,29,33] and another 30% (6/20) recorded type of milk [17,19,20,32,36,63]. The remaining DQI (*n* = 8, 32%) did not specify dairy or milk as low-fat.

Fourteen of the DQI included points for alcohol [11,16,17,18,19,21,22,23,29,33,34,36,38,39], however there was not agreement on the level of intake that was recommended. Almost all of the DQI (*n* = 13), except ARFS, considered alcohol to be part of a positive diet quality, and nearly half the tools counted alcohol intake in terms of range [11,18,21,22,23,33]. Fluid intake, including water, tea and coffee, was measured in DQI (*n* = 10), such as HEIFA-2013 [16] and DGI-2013 [17]. Furthermore, all Australian Dietary Guideline-based indices (*n* = 12) were composed of discretionary foods; 50% of them (6/12) included jam, ice-cream or chocolate [16,19,29,36,38,42], while 33% (4/12) included salt [17,18,19,36], sugar [16,17,18,33], confectionary [16,19,36,38] and sugar-sweetened beverages [16,19,36,38]. The remaining 17% (2/12) included take away food [38] or meat products [18,38] or fried food [36,38] or non-specified extra food [19,68] or extra ratios [30].

The most commonly found macronutrient in DQI was fat. Twenty percent (*n* = 5) of the DQI [8,16,18,33,34] were composed of saturated fatty acid (SFA); 12% (*n* = 3) were composed of trans-fatty acids [22,23,34] or PUFA [16,22,34]; 8% (*n* = 2) were composed of MUFA [16,34], total fat [8,34] or cholesterol [8,34]. In addition, fat ratio was included in 4 DQI; such as the ratio of MUFA to SFA [21,47], ratio of PUFA to SFA [23] and proportion of unsaturated fats to SFA [30]. Protein was included in two DQI such as DII [34] and ADQS [30]. Moreover, micronutrients such as sodium (*n* = 4) [15,16,18,22], other minerals (*n* = 2) [8,34] and vitamin (*n* = 1) [34] were included as components of DQI.

In addition to food and nutrients, 36% (9/25) of the DQI included dietary variety or diversity [8,16,17,18,19,28,33,37,38]; and one DQ index (4%) included dietary moderation as a separate component [8]. Lifestyle behaviors, such as physical activity [33] and multivitamin use [23], were also included. Cooking practices such as salt use (*n* = 6) and trimming of fat either before or after cooking (*n* = 7) were considered in the development of DQ indices. Likewise, eating patterns such as fast food or takeaway consumption (*n* = 3) [20,32,38], breakfast consumption (*n* = 1) [20] and ratio of energy intake to energy expenditure (*n* = 1) [33] were included.

### 3.3. Scoring of the Diet Quality Indices

The items within each DQ index were typically classified as recommended to be included as part of a healthy diet, or to be limited. Some indices assessed DQ by including foods that are recommended as well as those that should be limited (*n* = 21), while others focused on recommended foods only (*n* = 3) [29,41,42], or foods to limit only (*n* = 1) [31] (refer to Table S3). The items included in the DQI were quantified with different measurement units such as servings [8,15,16,17,18,19,20,22,23,28,32,33,36,37,38], grams [11,18,21,22,23,30,33,39,47], milligrams [15,16,18,22,33], standard drinks [22,36], proportions [16,17,19,30,36,37,38], % energy [8,16,18,22,23,33], % recommended dietary allowance (% RDA) [8], ratio [21,23,33,47], type of food item [17,19,20,28,32,36,38], frequency [16,17,19,20,28,32,36,38], reported consumption [29,31,41,42], mean (sd) value from global database [34] and kilo Joules [30].

For scoring, 13 out of 25 DQI (52%) used specific cutoffs for minimum and maximum intakes for each component and then calculated the intermediate proportional score [8,16,17,18,19,22,23,28,32,33,36,37,38]; five DQI (25%) used median or quintile intakes of the sample [11,15,21,39,47]; four DQI used the reported consumption of any amount of the component, or alternatively no consumption [29,31,41,42]; one DQ index based on the effect of the food parameter on inflammation [34], one DQ index based on DHQ responses [20], one DQ index by combining recommended dietary intake (RDI) and mean intake [30].

### 3.4. Dietary Assessment Methods Used

Generally, in nutritional epidemiology studies, dietary intake was measured by using real-time recording methods such as (weighted) food record and duplication method, and recall methods such as diet history, food frequency questionnaire (FFQ) and 24-h recall (24-h R) [69]. Food frequency questionnaire alone was the predominant method of dietary assessment in derivations of DQI (*n* = 13). Together with FFQ other dietary assessment methods were used in validation studies of DQI; 24-h Rs [18,19,39]; and weighed food records (WFRs) [16,31]. Short dietary assessment tools were also used in DQI development and/or evaluation; 38-item short food survey (SFS) [38], 24-item short dietary question (12-item FFQ and 12-item dietary behavior questions) [36] and 13-item question [32]. Furthermore, a 25-item dietary habits questionnaire and multiple-pass 24-h R were used in HDHI [20].

### 3.5. Evaluation of Diet Quality Indices

The DQI were evaluated in various ways. Reproducibility, the index’s ability to yielding similar outcomes on two different occasions [42] and reliability and internal consistency [28,63] were assessed. Content validity, which is the ability of DQ index items to reflect all contents or aspects it is supposed to measure [20], and construct validity by exploring the relationships between DQI and sociodemographic, health and behavioral characteristics, food and nutrient intakes [17,18,19,20,28,29,32,66] was also evaluated. Additionally, relative or criterion validity was evaluated by investigating the agreement of DQ scores between two different dietary assessment methods and predicting mortality or morbidity [8,16,18,20,32,42,63,68].

### 3.6. Summary Findings of Studies Investigating Diet Quality Indices, Health Outcomes and Non-Health Related Factors

Table 2 summarizes the major finding of the publications that used the DQI. The most frequently used DQI are ARFS (*n* = 12) [29,70,71,72,73,74,75,76,77,78,79,80], DGI-2013 (*n* = 11) [17,81,82,83,84,85,86,87,88,89,90], TDS (*n* = 10) [33,68,91,92,93,94,95,96,97,98,99], DGI (*n* = 9) [19,70,91,100,101,102,103,104,105], DII (*n* = 9) [106,107,108,109,110,111,112,113,114] and Mediterranean diet-based indices (*n* = 8) [71,72,88,89,106,107,108,115]. Some studies (*n* = 9) used two or more DQI in observing association between DQ and health related outcomes [70,71,72,84,88,89,106,107,108]. Health related outcomes or measurements or biomarkers observed in the articles using reviewed DQI were anthropometric measurements (*n* = 8); depression (*n* = 5); diabetes mellitus or abnormal glucose metabolism (*n* = 4); cardiometabolic risk factor or hypertension (*n* = 5), mortality (*n* = 3), cancer (*n* = 3), overweight or obesity (*n* = 3), quality of life or functional status or psychological function (*n* = 3), telomere length or aging (*n* = 2), sensory impairment (*n* = 2), vascular dysfunction (*n* = 2), chronic kidney disease (*n* = 1), asthma (*n* = 1) and inflammatory marker (*n* = 1).

The studies reported that there were relationships between high DQ scores and favorable health related outcomes. Negative associations were found between high DQ and all-cause mortality (HR_Q5_ vs. _Q1_:0.79; 95% CI: 0.63, 0.98; P_trend_ = 0.04) [33], total mortality (HR_Q5_ vs. _Q1_:0.86; 95% CI: 0.80–0.93; P_trend_ < 0.0001) [108] and cancer mortality (HR_T3_ vs. _T1_:0.3; 95% CI: 0.11, 0.83; P_trend_ = 0.06) [18]. However, there was inconsistent findings for associations between DQ and risk of overweight or obesity [74,75,76]; diabetes mellitus or abnormal glucose metabolism [70,97,100,116]; cancer [106,107,110]; and depression [30,72,73].

Non-health related factors associated with DQ were sociodemographic characteristics such as sex [19,36,117], age [19,20,35,77,101,117], education [17,77,103], occupation [17,86,101], income [19,103], socioeconomic status [19,20,29,37,83,103,117] and residence [17]. Moreover, lifestyle factors such as smoking [17,19,20,36,117], alcohol consumption [20], physical activity [17,36,77,101], nutrition knowledge [80,118], cooking meals for oneself [91], number of meals shared [35], meal frequency [81] and takeaway meal consumption [91,119] were found to be associated with DQ.

**Table 2:** Summary of findings from studies investigating diet quality indices, health outcomes and non-health-related factors.

Index	Reference	Validation Status of Diet Quality Index	Population Used in	Dietary Assessment Methods Used in Publications	Health-related Outcomes	Summary of Findings
Australian Healthy Eating Index (Aust-HEI)	Forsyth, 2012 [120] Forsyth, 2015 [121]	Tested construct validity [28]	Adults aged ≥18 years with depression and anxiety [120,121]	Diet History Questionnaire [120,121]	Depression, Anxiety and Stress Scale (DASS) [120,121]	Mean total Aust-HEI was 42.8 (range 20–60), and Aust-HEI and DASS were negatively correlated (*p* < 0.001) [120]. Improved DASS in the diet and physical activity intervention group (*p* < 0.05) [121].
Australian Recommended Food Score (ARFS)	Collins, 2008 [29] Collins, 2011 [79] Morrison, 2012 [77] Aljadani, 2013 [74] Aljadani, 2013 [75] Alhazmi, 2014 [70] Potter, 2014 [78] Petersen, 2015 [71] Aljadani, 2016 [76] Kullen, 2016 [80] Lai, 2016 [72] Lai, 2017 [73]	Tested construct validity [29]	Adults aged ≥50 years [71]; mid-aged women (50–55 years) [29,70,72,73,74,76,78,79]; young women (mean age: 27.6 ± 1.5 years and 34.2 ± 5.1 years) [75,77]; young men (mean age: 28.7 ± 8.9 years) [80]	FFQ (74-item food and 6-item alcohol) [29,70,71,72,73,74,75,76,77,78,79,80]	Diabetes [70]; Depression [72,73]; Overweight or obese [74,75,76]; Diet quality [29,71,77,78,79,80]	No association between ARFS and diabetes risk [70]. Women who maintained moderate or high ARFS scores had a low risk of depression (*p* = 0.045 and 0.01) [73], but no longitudinal association between ARFS and depressive symptoms [72]. Association between ARFS and overweight or obesity is not consistent [74,75,76]. Factors associated with higher ARFS were socioeconomic status, education, marital status, smoking, physical activity (all *p* < 0.0001) [29]; age, education, physical activity (all *p* < 0.001) [77]; nutrition knowledge (*p* = 0.009) [80].
Australian Recommended Food Score-1 (ARFS-1)	Baker, 2014 [122] O’ Brien, 2014 [123] Collins, 2015 [42] Ashton, 2017 [124] Ashton, 2017 [125] Williams, 2017 [35] Ashton, 2018 [126] Harbury, 2019 [118]	Tested reproducibility, comparative validity [42]; relative validity [124]	Adults aged ≥16 years [35], ≥18 years [118,122,123,124,126], ≥30 years [42]; young men aged 18–25 years [125]	Subset of 70 items from 120-item FFQ [42,118,122,123,124,125,126]; Healthy Eating Quiz (online survey, 70 items) [35]	Plasma carotenoid [124]; Weight loss [123]; Diet quality [42,118,122,125,126].	Significant correlation between total ARFS-1 and plasma total carotenoids (r = 0.17, *p* < 0.05) [124]. The intervention groups significantly lost more weight than the control group after 12-weeks (*p* < 0.001) [123]. Factors associated with ARFS-1 were nutrition knowledge and BMI (*p* < 0.001) [118].
Dietary Guideline Index (DGI)	McNaughton, 2008 [19] McNaughton, 2009 [100] Arabshahi, 2011 [101] Arabshahi, 2012 [102] Thorpe, 2013 [91] Alhazmi, 2014 [70] Backholer, 2016 [103] Olstad, 2017 [104] Smith, 2017 [105]	Tested construct validity [19]	Adults aged ≥19 years [19], 18–36 years [91], ≥25 years [100,101,102,103], 26–36 years [105]; mid-aged women (50–55 years) [70], women 18–46 years [104]	FFQs: 74-item [100], FFQ (74-item food and 6-item alcohol) [70,103], 107-item [91], 151-item [101,102], items not mentioned [104]; FFQ and others: 127-item FFQ and food habit questionnaire (FHQ) [105]; 108-item FFQ, single 24-h R [19]	Diabetes [70,100] and cardiometabolic risk factors [100]; Anthropometric measurements [102,104]; Diet quality [19,91,101,103,105]	DGI was negatively associated with diabetes in men (OR_Q4-Q1_:0.38, 95% CI: 0.18–0.80) [100] and women (OR_Q5-Q1_:0.51; 95% CI: 0.35, 0.76) [70]; hypertension in both sexes (OR_Q4-Q1_:0.5, 95% CI: 0.31–0.81) [100]. Association between DGI and waist circumference (WC) [100,102]; body mass index (BMI) [102,104] was inconsistent. Factors associated with DGI were sex (*p* < 0.05) [19], age (both *p* < 0.05) [19,101], education (*p* < 0.01) [103], income (*p* < 0.05, <0.01) [19,103], socioeconomic status (*p* < 0.05, <0.01) [19,103], smoking (*p* < 0.05) [19], physical activity (both *p* < 0.05) [19,101], occupation (*p* < 0.05) [101], hormonal replacement therapy (*p* < 0.05) [101], cooking meals for oneself (*p* = 0.001) [91], and takeaway and convenient meal consumption (*p* < 0.001) [91].
Modified Dietary Guideline Index (Modified DGI)	McLeod, 2011 [37]	Not tested	Women (mean age = 32.3 years) [37]	137-item FFQ [37]	Diet quality [37]	Diet quality was significantly better in women of a high socioeconomic group as compared to those of the low socioeconomic group (*p* < 0.001) [37].
Dietary Guideline Index-2013 (DGI-2013)	Milte et al. 2015 [89] Livingstone, 2016 [84] Thorpe, 2016 [17] Leech, 2016 [81] Leech, 2017 [82] Livingstone, 2017 [83] Martin, 2017 [86] Ribeiro, 2017 [90] Livingstone, 2018 [85] Milte, 2018 [88] Martin, 2019 [87]	Tested construct validity [17]	Adults aged ≥19 years [81,82,83,84,85], 55–68 years [17,88,89]; women aged 18–50 years [86,87]; men aged ≥74 years [90]	FFQ (74-item food and 6-item alcohol) [86,87], 111-item FFQ and food-related behavior questions [17,88,89]; two 24-h Rs [81,82,83,84,85]; diet histories questionnaire [90]	Obesity [82,84,85,90] Hypertension [84]; health related quality of life (QOL) [89]; Telomere length [88]; Diet quality [17,81,83,86,87]	Higher DGI-2013 scores were negatively associated with obesity measured by BMI (both P_trend_ < 0.05) [84,85], WC (both P_trend_ < 0.05) [84,85], waist–hip ratio (WHR) (*p* < 0.001) [90]. Men with higher DGI-2013 were less likely to be hypertensive (P_trend_ < 0.05) [84]. Higher DGI-2013 scores were associated with better health-related QOL (*p* < 0.05) [89]. No association between DGI-2013 and relative telomere length [88]. Factors associated with DGI-2013 were sex (*p* < 0.001), residence (men, *p* < 0.001) [17], occupation (men: *p* = 0.02; women: *p* = 0.043) [17,86], income (women: *p* = 0.013) [86], education (*p* < 0.001) [17], socioeconomic status (P_trend_ < 0.001) [83], smoking (*p* < 0.001) [17], physical activity (*p* < 0.001) [17], BMI (*p* < 0.001) [17], frequency of meals (*p* < 0.001) [81].
RESIDential Environments (RESIDE) Dietary Guideline Index (RDGI)	Bivoltsis, 2018 [36]	Not tested	Adults aged ≥25 years [36]	24-item questionnaire (12 from validated FFQ, 12 from validated dietary behavior questions) [36]	Diet quality [36]	Two simple RESIDE dietary guideline indices using subsets of six survey items (S-RDGI1), and nine survey items (S-RDGI2) showed reasonable agreement with RDGI (Spearman rho = 0.78, 0.84). For all indices, higher diet quality was associated with sex (all *p* < 0.001), age (S-RDGI1 and S-RDGI2, *p* < 0.001), smoking status (S-RDGI1: *p* = 0.001, SRDGI and S-RDGI2: *p* < 0.001), physical activity (RDGI: *p* = 0.001, S-RDGI1: *p* < 0.0001, S-RDGI2: *p* = 0.002) [36].
Commonwealth Scientific and Industrial Research Organization Healthy Diet Score (CSIRO HDS)	Hendrie, 2017 [63] Hendrie, 2017 [38] Hendrie, 2018 [127]	Tested reliability and relative validity [63]	Adults aged ≥18 years [38,127], aged 19–50 years [63]	38-item SFS [38,127]; 38-item SFS and three 24-h Rs [63]	Obesity [127]; Diet quality [38,63]	Adults having a lower score were more likely to obese (OR_Q1-Q5_ 2.99, CI: 2.88, 3.11) [127]. Women scored higher than men (59.9 ± 12.6 vs. 56.2 ± 13.1), older adults higher than younger adults (>71yr: 63.1 ± 11.7 vs. 18–30 yr: 57.3 ± 13.2), and normal-weight adults higher than obese adults (60.5 ± 12.6 vs. 55.7 ± 13.2) [38].
Total Diet Score (TDS)	Russell, 2013 [33] Gopinath, 2013 [96] Gopinath, 2013 [95] Gopinath, 2013 [97] Gopinath, 2014 [98] Gopinath, 2014 [92] Hong, 2014 [94] Gopinath, 2016 [93] Roach, 2017 [99] Russell, 2017 [68]	Tested criterion validity [33]	Adults aged ≥49 years [33,93,94], ≥50 years [95,96,97,98], ≥55 years [92], 65–85 years [68], median age-72 years [99]	145-item FFQ [33,92,93,94,95,96,97,98], 145-item FFQ and 4-day WFRs [68], three 24 h Rs and PUFA FFQ [99]	All-cause mortality [33]; Chronic kidney disease (CKD) [96], visual impairment [94], retinal vascular change [95], quality of life (QOL) [92], aging [93] Impaired fasting glucose (IFG) and diabetes [97], dual sensory impairment (DSI) [98], Diet quality [68,99]	Those in the highest TDS quintile had reduced risk of all-cause mortality (P_trend_ = 0.04) [33]. Those in highest TDS quartile had reduced risk of CKD (P_trend_ = 0.005) [96], reduced risk of visual impairment (>65yrs: *p* = 0.05) [94], healthier retinal vessels (P_trend_ < 0.05), but not associated with 5-y change in retinal vessel caliber [95], good QOL (P_trend_ < 0.05) [92] and successful aging (OR: 1.58, 95% CI: 1.02, 2.46) [93]. Negative association between high TDS and risk of IFG in men (P_trend_ = 0.02), but no association in women for diabetes risk [97]. No association between baseline TDS and DSI [98]. No significant mean TDS difference between results from FFQ and WFR (*p* = 0.63), but significant correlation between the two methods (r = 0.75, *p* < 0.0001) [68].
Aussie-Diet Quality Index (Aussie-DQI)	Zarrin, 2013 [18]	Tested content, construct and criterion validity [18]	Adults aged ≥19 years from 1995 National Nutrition Survey (NNS); aged ≥25 from the Nambour Skin Cancer Study (NSC) [18]	129-item FFQ and a 24-h R [18]	All-cause and cancer mortality [18]	Higher Aussie-DQI scores were associated with higher desirable nutrient intakes and inversely associated with risk of cancer mortality in men (HR: 0.3, 95% CI: 0.11, 0.83) [18].
Healthy Eating Index for Australian Adults-2013 (HEIFA-2013)	Roy, 2016 [16] Roy, 2017 [119] Grech, 2017 [117] Grech, 2017 [128]	Tested criterion validity and internal consistency [16]	Adults aged 18–34 years [16,117,128], 19–24 years [119]	FFQ (74-item food and 6-item alcohol) and 5-d WFR [16], validated mobile application (e-DIA app) [119], two 24-h Rs [117,128]	Diet quality [16,117,119]; Dietary energy density [128]	Positive correlation of essential micronutrients between both FFQ and WFR HEIFA-2013 scores (P_trend_ < 0.0005, Cronbach α = 0.41) [16]. Higher HEIFA-2013 was associated with reduced university campus and other takeaway foods consumption (P_trend_ < 0.001), BMI (P_trend_ = 0.02) and WC (P_trend_ = 0.05) [119]; sociodemographic and lifestyle characteristics (*p* < 0.05) [117]. Higher dietary energy density was associated with lower HEIFA-2013 (*p* < 0.0001) [128].
Australian Diet Quality Score (ADQS)	Froud, 2019 [30]	Not tested	Adults aged 18–75 years [30]	FFQ (74-item food and 6-item alcohol) [30]	Depression [30]	Lower ADQS was associated with increased depression risk (*p* = 0.037) [30].
Healthy Dietary Habits Index (HDHI)	Wong, 2017 [20] Davison, 2017 [129]	Tested content, construct and criterion validity [20]	Adults aged ≥19 years [20], child–parent pairs (mean age of child = 10.2 years, parent = 41.6 years) [129]	Two 24-h Rs and 25-item DHQ [20], Children; 28-item FFQ and Parents; 25-item DHQ [129]	Diet quality [20,129]	Higher HDHI score was associated with sociodemographic and lifestyle characteristics; higher nutrient intakes (all *p* < 0.001) [20]. Parental DQI score was associated with a child’s dietary pattern score (*p* < 0.001) [129].
Diet Quality Index-Revised (DQI-R)	Reeves et al. 2013 [116]	Tested reproducibility and validity [57]	Adults aged ≥25 years [116]	74-item FFQ [116]	AGM- Abnormal glucose metabolism (IFG, impaired glucose tolerance, diabetes) [116]	Women with low DQI-R were more likely to have AGM (P_trend_ = 0.012) [116].
Recommended Food Score (RFS)	Milte et al. 2015 [89] Livingstone, 2016 [84] Milte, 2018 [88]	Not tested	Adults aged 55–68 years [88,89], ≥19 years [84]	111-item FFQ and food-related behavior questions [88,89], two 24-h Rs [84]	Health-related QOL [89]; obesity and hypertension [84]; Telomere length [88]	Higher RFS scores were associated with better health-related QOL (P_trend_ < 0.001) [89] and less likely to be hypertensive (P_trend_ = 0.021) [84]. No association between RFS and telomere length [88].
Not Recommended Food Score (NRFS)	Petersen, 2015 [71]	Not tested	Adults (mean age = 50 years) [71]	FFQ (74-item food and 6-item alcohol) [71]	Diet quality [71]	Mean NRFS scores for participants with diabetes and controls were not different [71].
Mediterranean Diet Score (MD Score)	Petersen, 2015 [71] Dugue, 2016 [106] Hodge, 2016 [107] Hodge, 2018 [108]	Not tested	Adults (mean age = 50 years) [71], aged 27–76 years [106], mid-aged adults 40–69 years [107,108]	FFQ (74-item food and 6-item alcohol) [71], 121-item FFQ [106,107,108]	Urothelial cell carcinoma (UCC) incidence [106]; lung cancer [107]; total, cardiovascular disease (CVD), coronary heart disease (CHD) mortality [108]; Diet quality [71]	Higher MD score was inversely associated with invasive UCC (HR: 0.86; 95% CI: 0.74, 1.00) [106], lung cancer risk (HR_7-9_ vs. _0–3_:0.64; 95% CI: 0.45, 0.90) [107] and total mortality (HR_Q5-Q1_:0.86; 95% CI: 0.80, 0.93) [108]. Mean MD scores for participants with diabetes and controls were not different [71].
Mediterranean Diet Pattern index (MDP index)	Lai, 2016 [72]	Not tested	Mid-aged women (50–55 years) [72]	FFQ (74-item food and 6-item alcohol) [72]	Depressive symptoms [72]	Inverse association between MDP index and depressive symptoms (P_trend_ = 0.007) [72].
MedDiet Score	Crichton, 2013 [115]	Not tested	Adults aged 40–65 years [115]	215-item FFQ [115]	Self-reported psychological functioning [115]	Total MedDiet score was not associated with cognitive function, but plant food intakes was beneficial for general health and mental disorders (*p* < 0.05) [115].
Mediterranean Diet Scale (MDS)	Milte, 2015 [89] Milte, 2018 [88]	Not tested	Adults aged 55–68 years [88,89]	111-item FFQ and food-related behavior questions [88,89]	Health-related QOL [89]; Telomere length [88]	Higher MDS scores were associated with better health-related QOL (*p* < 0.001) [89]. No association between MDS and relative telomere length [88].
Dietary Approach to Stop Hypertension (DASH)	Petersen, 2015 [71]	Not tested	Adults (mean age = 50 years) [71]	FFQ (74-item food and 6-item alcohol) [71]	Diet quality [71]	Mean DASH scores for participants with diabetes and controls were not different [71].
Alternative Healthy Eating Index (AHEI)	Petersen, 2015 [71]	Not tested	Adults (mean age = 50 years) [71]	FFQ (74-item food and 6-item alcohol) [71]	Diet quality [71]	Mean AHEI scores for participants with diabetes and controls were not different [71].
Alternative Healthy Eating Index-2010 (AHEI-2010)	Dugue, 2016 [106]	Not tested	Adults aged 27–76 years [106]	121-item FFQ [106]	Urothelial cell carcinoma (UCC) incidence [106]	No association between AHEI-2010 and risk of overall UCC (HR: 1.03; 95% CI: 0.92, 1.15) and invasive UCC (HR: 0.88; 95% CI: 0.75, 1.04) [106].
Diet Quality Tool (DQT)	O’Reilly, 2012 [32]	Tested construct and criterion validity [32]	CVD patients (mean age = 61.2 ± 10.8 years) [32]	13-item questionnaire from validated FFQ and 4-d food diary [32]	Diet quality [32]	Significant difference was found between mean dietary fiber (*p* < 0.05) and% total energy from saturated fat (*p* < 0.01) for those with better DQT scores (>60%) vs. poorer scores (≤60%) when compared with 4-day food diary nutrient values [32].
Dietary Inflammatory Index (DII)	Wood, 2015 [114] Dugue, 2016 [106] Hodge, 2016 [107] Shivappa, 2016 [111] Vissers, 2016 [113] Vissers, 2017 [112] Hodge, 2018 [108] Mayr, 2018 [109] Nagle, 2019 [110]	Tested construct validity [66]	Adults aged ≥18 years [110,114], 27–76 years [106], mid-aged adults 40–69 years [107,108]; mid-aged women (50–55 years) [111,112,113]; mean age-61.9 years [109]	FFQ (74-item food and 6-item alcohol) [111,112,113], 121-item FFQ [106,107,108], 139-item FFQ [110], 186-item FFQ [114], 7-day food diary [109]	lung cancer [107]; total, CVD, CHD mortality [108]; ovarian cancer risk and survival [110]; hypertension [112]; CVD, CHD and cerebrovascular disease risk [113]; Asthma risk [114]; Interleukin 6 (IL-6) [109]; depression [111]; UCC incidence [106]	Higher DII score (pro-inflammatory diet) was positively associated with risk of total mortality (HR_Q5-Q1_:1.16; 95% CI: 1.08, 1.24) [108]; lung cancer in current smokers (HR_Q4-Q1_:1.70; 95% CI: 1.02, 2.82) [107]; ovarian cancer (OR_Q4-Q1_:1.31; 95% CI: 1.06, 1.63) [110]; hypertension (OR: 1.24; 95% CI: 1.06, 1.45) [112]; myocardial infarct (HR: 1.46; 95% CI: 1.12, 1.89) [113] and asthma (OR: 1.70; 95% CI: 1.03, 2.14) [114]. Lower DII score (anti-inflammatory diet) was negatively associated with depression (RR_Q1-Q4_:0.81, 95% CI: 0.69, 0.96) [111] and high sensitivity IL-6 (r = 0.34, 95% CI: 0.05, 0.56) and triglyceride (r = −0.30, 95% CI: −0.51, −0.06) [109]. No association between DII and risk of overall UCC (HR: 1.06; 95%CI: 0.96, 1.18) [106].

### 3.7. Critical Appraisal of Diet Quality Indices by Previous Suggested Recommendations

The DQI [8,11,15,16,17,18,19,20,21,22,23,28,29,30,31,32,33,34,36,37,38,39,41,42,47] were described and shaded gray according to their adherence to the recommended points developed by Burggraf et al. and Trijsburg et al. (Table 3). Sixteen of the 25 DQI were constructed to reflect the respective dietary guidelines, and the remaining indices measured condition-specific recommendations. Almost all the DQI (*n* = 21), except ARFS, ARFS-1, RFS and NRFS, captured the dimensions of adequacy (foods that people should eat more of) and moderation (foods that people should limit). Dietary variety was considered in almost half of the indices (*n* = 12). Nearly one-fourth of the indices included the dimensions of adequacy, moderation and balance [11,21,22,23,30,47]. With respect to the dimensional structure, only five indices had a nested structure [16,17,19,33,36], but ten indices had an ordered structure [8,18,21,29,37,39,41,42,47,63]. As database or dietary assessment method for construction of DQI, food frequency questionnaires, either alone or with other dietary assessment methods, were mostly used (*n* = 20). Twelve indices consisted of foods, food groups and nutrients [8,11,15,16,18,21,22,23,30,33,34,47]. The DQI used different scoring systems; metric (*n* = 11), dichotomous (*n* = 8) and ordinal (*n* = 6). Most of the DQI with metric scales used normative cutoff for minimum and maximum intakes for each component and then calculated the intermediate proportional score, for example, DGI, Aussie-DQI, AEHI-2010. Based on evaluation of DQI, construct validity [17,18,19,20,28,29,32,66], relative validity [8,16,18,20,32,42,63,68], content validity [20], reproducibility [42], reliability and internal consistency [28,63] were reported.

**Table 3:** Critical appraisal of diet quality indices by previously suggested recommendations.

	Theoretical Framework		Dimension				Structure	Indicator Selection			Scoring Criteria			Aggregation	
	Dietary Guideline	Dietary Pattern	Adequacy	Moderation	Variety	Balance	Nested/ Ordered/ Not Ordered	Database	Foods & Food Groups/ Nutrients/ Both	Healthy/ Unhealthy Component	Dichotomous/ Ordinal/ Metric	Range	Cut Points	Weighted Equally by Indicators	Evaluation of DQI
Aust-HEI [28]	Y		Y	Y	Y		Not ordered	FFQ (item not stated), SDQ	Foods & food groups	Y	Ordinal	[0, 60]	Y	Y	Construct Validity
ARFS [29]	Y				Y		Ordered	FFQ (74-item food and 6-item alcohol)	Foods & food groups	N	Dichotomous	[0, 74]	N	Y	Construct Validity
ARFS-1 [42]	Y				Y		Ordered	Subset of 70 items from 120-item FFQ	Foods & food groups	N	Dichotomous	[0, 73]	N	Y	Reproducibility, comparative validity
DGI [19]	Y		Y	Y	Y		Nested	108-item FFQ, Single 24-h R	Foods &food groups	Y	Metric	[0, 150]	Y	Y	Construct Validity
Modified DGI [37]	Y		Y	Y	Y		Ordered	137-item FFQ	Foods & Food groups	Y	Metric	[0, 80]	Y	Y	Not tested
DGI-2013 [17]	Y		Y	Y	Y		Nested	111-item FFQ	Foods & food groups	Y	Metric	[0, 130]	Y	Y	Construct Validity
RDGI [36]	Y		Y	Y			Nested	12-item FFQ, 12-item DBQ	Foods & food groups	Y	Metric	[0, 100]	Y	Y	Not tested
CSIRO HDS [63]	Y		Y	Y	Y		Ordered	38-item SFS	Foods & food groups	Y	Metric	[0, 100]	Y	Y	Relative validity
TDS [33]	Y		Y	Y	Y		Nested	145-item FFQ	Both	Y	Ordinal	[0, 20]	Y	Y	Relative validity
Aussie-DQI [18]	Y		Y	Y	Y		Ordered	Single 24-h R, 129-item FFQ	Both	Y	Metric	[0, 120]	Y	Y	Construct Validity, criterion validity
HEIFA-2013 [16]	Y		Y	Y	Y		Nested	Five 1-day WFR, FFQ (74-item food and 6-item alcohol)	Both	Y	Ordinal	[0, 100]	Y	Y	Internal consistency, relative validity
ADQS [30]	Y		Y	Y		Y	Not ordered	FFQ (74-item food and 6-item alcohol)	Both	Y	Metric	Maximum = RDI(−10%)	Y	Y	Not tested
HDHI [20]	Y		Y	Y			Not Ordered	Multiple-pass single 24-h R, 25-item DHQ	Foods & food groups	Y	Ordinal	[0, 60]	Y	Y	Content validity, construct validity, Relative validity
DQI-R [8]	Y		Y	Y	Y		Ordered	Two 24-h Rs	Both	Y	Metric	[0, 100]	Y	Y	Concurrent validity
RFS [41]	Y		Y		Y		Ordered	23 items from 62-item FFQ	Foods & food groups	N	Dichotomous	[0, 23]	N	Y	Not tested
NRFS [32]	Y			Y			Not ordered	60-item FFQ, WFRs (days not stated)	Foods & food groups	N	Dichotomous	[0, 21]	N	Y	Not tested
MD score [11]		Y	Y	Y		Y	Not ordered	190-item FFQ	Both	Y	Dichotomous	[0, 8]	N	Y	Not tested
MDP index [21]		Y	Y	Y		Y	Ordered	150-item FFQ	Both	Y	Dichotomous	[0, 9]	N	Y	Not tested
MedDiet score [47]		Y	Y	Y			Ordered	121-item FFQ	Foods & food groups	Y	Dichotomous	[0, 9]	N	Y	Not tested
MDS [39]		Y	Y	Y		Y	Ordered	FFQ (item not stated), 24–h R (days not stated)	Both	Y	Dichotomous	[0, 9]	N	Y	Not tested
DASH [15]		Y	Y	Y			Not ordered	116-item FFQ	Both	Y	Ordinal	[8, 40]	N	Y	Not tested
AHEI [23]		Y	Y	Y		Y	Not ordered	130-item FFQ	Both	Y	Metric	[2.5, 87.5]	Y	Y *	Not tested
AHEI-2010 [22]		Y	Y	Y		Y	Not ordered	FFQ (item not stated)	Both	Y	Metric	[0, 110]	Y	Y	Not tested
DQT [32]		Y	Y	Y			Not ordered	4-day FD, 13-item questionnaire	Foods & food groups	Y	Ordinal	[0, 130]	Y	Y	Construct and criterion validity
DII [66]		Y	Y	Y	Y		Not ordered	7-day dietary recalls, 24-h Rs [67]	Both	Y	Metric	[−8.87, +7.98]	N	Y	Construct validity [67]

24-h R: 24-h recall; DBQ: dietary behavior questions; DHQ: dietary habit questionnaire; FD: food diary; FFQ: food frequency questionnaire; N: No; RDI: recommended daily intake; SDQ: short dietary questions; SFS: short food survey; WFR: weighted food record; Y: Yes. * valid for except multivitamin use. The preferable features of DQI in the table are highlighted.

## 4. Discussion

This systematic review and critical appraisal summarizes 25 DQI represented in 76 papers, including Australian and New Zealand adults. When the Australian and New Zealand DQI were assessed by the recommended points suggested by the previous reviews, none of them met all suggested criteria. Almost half of the reviewed DQI were composed of recommended foods or food groups and nutrients, and based on metric indicators, as is recommended by Burggraf et al. [14]. Nearly one-quarter of DQI included adequacy, moderation, and balance, key dimensions outlined by Burggraf et al. (2018). However, only one-third of the DQI were constructed according to the preferred nested structure, i.e., constructed with hierarchy, allowing for in-depth analysis. As for the strengths of all the reviewed Australian and New Zealand DQI, components were equally weighted by indicators, and their construct and criterion validity had been evaluated. DGI, DGI-2013, TDS, HEIFA-2013 and Aussie-DQI performed best according to dimension and its structure, indicator selection, scoring criteria and evaluation.

Our review combines the recommendations from Burggraf et al. (2018) and Trijsburg et al. (2019), each of whom presented an inventory of DQI construction criteria using a range of international DQI. Only 11 of the 25 DQI used in Australian and New Zealand adults included in our review overlapped with those selected in the previous reviews, making ours the most comprehensive review of DQI for this region. Like DQI applicable in LMIC [13], separate scoring for healthy and unhealthy items was not reported in DQI used in Australian and New Zealand adults. In addition, similar to Burggraf et al. [14], most of the DQI used in Australian and New Zealand adults were constructed by focusing on health outcomes linked with overconsumption or over-nutrition. As suggested by the recent systematic review [13], it would be beneficial to develop a global DQ index covering both over- and under-nutrition aspects for applying in different cross-countries settings.

Among the DQI based on Australian Dietary Guidelines, five indices were constructed with nested structure [16,17,19,33,36]; three indices is based on the latest Australian Dietary Guideline [16,17,36], three indices being composed of metric scaling [17,19,36] and evaluated among various age group (≥19 years in DGI and Aussie-DQI, 55–65 years in DGI-2013, 63–83 years in TDS, 18–34 years in HEIFA-2013). For example, both DGI-2013 and HEIFA-2013 had similarities in terms of dimension, dimensional structure, cutoff points and evaluation, but differences in indicator selection (foods and food groups in DGI-2013, a combination of nutrients, foods and food groups in HEIFA-2013) and scaling (metric in DGI-2013, ordinal in HEIFA-2013). It would be advantageous to employ these indices for investigating the overall DQ of adults in longitudinal settings, similar to the work of Sotos-Prieto and colleagues [130]. The authors investigated the relationship between mortality and DQ assessed by three indices, which were different in description, composition and construction criteria [130].

Another DQ index appropriate for application in diet-health relationship studies is the Aussie-DQI constructed by Zarrin et al. [18]. It is an extensively evaluated DQ index by using two independent data sets for its development and validation. The evaluation of construct validity showed that higher Aussie-DQI scores were associated with gender, age, smoking status and body mass index [18]. Further, the criterion validity assessment demonstrated that there was a negative association between Aussie-DQI scores and cancer mortality among men [18]. Although most of its scoring was based on earlier Australian Dietary Guidelines [59,60], recommendations from the World Health Organization, United Kingdom and the United States of America were applied for intakes of processed meat, sugar and saturated fat. It would be beneficial to investigate the predictive ability of Aussie-DQI for morbidity and mortality in large population-based cohort studies.

Among DQI measuring specific dietary pattern recommendations, those based on a traditional Mediterranean diet had shown that beneficial effects on cancer, cardiovascular diseases and diabetes [131,132,133]. Nevertheless, these indices seem not to conform to the preferable features of DQI. They were generally developed with no nested structure, detailed scoring range with percentile cutoff points, and not evaluated for construct and criterion validity. It is intriguing to construct a Mediterranean diet-based index that meets with methodological finesse and applies to nutritional epidemiological studies [14]. In contrast, the AHEI-2010, which scores foods and nutrients that prevent chronic diseases based on the Mediterranean diet [48,49,50,51,52], seems to be more appropriate for application in diet-health relationship studies. The AHEI-2010 had promising features for dimensions (adequacy, moderation and balance), indicator selection (composed of a combination of nutrients, foods and food groups, with healthy and unhealthy components), metric scaling with normative cutoff points [22].

This review identified the available DQI used with Australian and New Zealand adults. All included studies were those that described the development, evaluation or validation and application of each DQ index for measuring the overall DQ of adults, especially in various Australian and New Zealand settings. The strength of the current review is that it applies newly defined criteria to critically appraise all existing DQI used in Australian and New Zealand adults. Two independent reviewers assessed eligibility and methodological quality and extracted data for the identified DQI. However, a limitation of this review is the use of only one database. Relevant DQI may have been missed if the papers did not report the words used in our search strategy. To avoid missing relevant DQI, our electronic database search was complemented by prescreening in a second database (EMBASE) and hand searching the reference lists of the retrieved papers, which yielded only one additional paper.

## 5. Conclusions

The development and application of DQI based on national nutritional guidelines, condition-specific recommendations or composition are expanding rapidly. Importantly, national dietary guidelines are updated periodically according to evidence-based information; and hence the development of DQI should be based on the recent dietary guidelines in order to capture the recent updates. Preferable features of DQ index such as theoretical framework, dimension, dimensional structure, indicator selection, scoring criteria, aggregation and its evaluation should be considered when applying DQI in diet-health relationship studies. While further work is needed to enhance the construction of all Australian and New Zealand DQI to bring them into alignment with recommended construction criteria, DGI, DGI-2013, TDS, HEIFA-2013 and Aussie-DQI performed relatively well.

## Supplementary Materials

The following are available online at https://www.mdpi.com/2072-6643/12/12/3777/s1, Table S1 Quality assessment of studies (ADA Quality Criteria Checklist Primary Research); Table S2 Overview of components of diet quality indices; Table S3 Components and scoring of diet quality indices.
