# Supplementary material for: Diet Quality Indices Used in Australian and New Zealand Adults: A Systematic Review and Critical Appraisal
Source: Nutrients. 2020 Dec 9;12(12):3777. doi: 10.3390/nu12123777 (PMC7763901; doi:10.3390/nu12123777)
Supplement: Supplementary file 1 [file nutrients-12-03777-s001.pdf]

Supplementary tables

Table S1 Quality assessment of studies (ADA Quality Criteria Checklist Primary Research)

Citation	Clear research question?	Free of selection bias?	Study groups comparable?	Method of handling withdrawals described?	Blinding?	Intervention described?	Clear outcomes, valid & reliable measurements?	Appropriate statistical analysis	Conclusions supported by results?	Bias due to funding? Sponsorship unlikely?	(-) or (ϕ) or (+)
Alhazmi, 2014 [1]	Y	Y	Y	NA	N	Y	Y	Y	Y	Y	(+)
Aljadani, 2013 [2]	Y	Y	Y	NA	N	Y	Y	Y	Y	Y	(+)
Aljadani, 2013 [3]	Y	Y	Y	Y	N	Y	Y	Y	Y	Y	(+)
Aljadani, 2016 [4]	Y	Y	Y	NA	N	Y	Y	Y	Y	Y	(+)
Arabshahi, 2011 [5]	Y	Y	Y	NA	N	Y	Y	Y	Y	Y	(+)
Arabshahi, 2012 [6]	Y	Y	Y	NA	N	Y	Y	Y	Y	Y	(+)
Ashton, 2017 [7]	Y	Y	Y	Y	Y	Y	Y	Y	Y	Y	(+)
Ashton, 2017 [8]	Y	Y	Y	NA	N	Y	Y	Y	Y	Y	(+)
Ashton, 2018 [9]	Y	Y	Y	NA	N	Y	Y	Y	Y	Y	(+)
Backholer, 2016 [10]	Y	Y	Y	NA	N	Y	Y	Y	Y	Y	(+)
Baker, 2014 [11]	Y	Y	Y	NA	N	Y	Y	Y	Y	Y	(+)
Bivoltsis, 2018 [12]	Y	Y	Y	NA	N	Y	Y	Y	Y	Y	(+)
Collins, 2008 [13]	Y	Y	Y	Y	N	Y	Y	Y	Y	Y	(+)
Collins, 2011 [14]	Y	Y	Y	Y	N	Y	Y	Y	Y	Y	(+)
Collins, 2015 [15]	Y	Y	Y	NA	N	Y	Y	Y	Y	Y	(+)
Crichton, 2013 [16]	Y	Y	Y	Y	N	Y	Y	Y	Y	Y	(+)
Davison, 2017 [17]	Y	Y	Y	NA	N	Y	Y	Y	Y	Y	(+)
Dugue, 2016 [18]	Y	Y	Y	NA	N	Y	Y	Y	Y	Y	(+)
Forsyth, 2012 [19]	Y	Y	Y	NA	N	Y	Y	Y	Y	Y	(+)
Forsyth, 2015 [20]	Y	Y	Y	Y	N	Y	Y	Y	Y	Y	(+)
Froud, 2019 [21]	Y	Y	Y	NA	N	Y	Y	Y	Y	Y	(+)
Gopinath, 2013 [22]	Y	Y	Y	NA	N	Y	Y	Y	Y	Y	(+)
Gopinath, 2013 [23]	Y	Y	Y	NA	N	Y	Y	Y	Y	Y	(+)
Gopinath, 2013 [24]	Y	Y	Y	NA	N	Y	Y	Y	Y	Y	(+)
Gopinath, 2014 [25]	Y	Y	Y	NA	N	Y	Y	Y	Y	Y	(+)
Gopinath, 2014 [26]	Y	Y	Y	NA	N	Y	Y	Y	Y	Y	(+)
Gopinath, 2016 [27]	Y	Y	Y	NA	N	Y	Y	Y	Y	Y	(+)
Grech, 2017 [28]	Y	Y	Y	Y	N	Y	Y	Y	Y	Y	(+)
Grech, 2017 [29]	Y	Y	Y	Y	N	Y	Y	Y	Y	Y	(+)

Quality assessment of studies (ADA Quality Criteria Checklist Primary Research)

Citation	Clear research question?	Free of selection bias?	Study groups comparable?	Method of handling withdrawals described?	Blinding?	Intervention described?	Clear outcomes, valid & reliable measurements?	Appropriate statistical analysis	Conclusions supported by results?	Bias due to funding? Sponsorship unlikely?	(-) or (ϕ) or (+)
Harbury, 2019 [30]	Y	Y	Y	Y	N	Y	Y	Y	Y	Y	(+)
Hendrie, 2017 [31]	Y	Y	Y	Y	N	Y	Y	Y	Y	Y	(+)
Hendrie, 2017 [32]	Y	Y	Y	Y	N	Y	Y	Y	Y	Y	(+)
Hendrie, 2018 [33]	Y	Y	Y	Y	N	Y	Y	Y	Y	Y	(+)
Hodge, 2016 [34]	Y	Y	Y	Y	N	Y	Y	Y	Y	Y	(+)
Hodge, 2018 [35]	Y	Y	Y	Y	N	Y	Y	Y	Y	Y	(+)
Hong, 2014 [36]	Y	Y	Y	Y	N	Y	Y	Y	Y	Y	(+)
Kullen, 2016 [37]	Y	Y	Y	N	N	Y	Y	Y	Y	Y	(+)
Lai, 2016 [38]	Y	Y	Y	Y	N	Y	Y	Y	Y	Y	(+)
Lai, 2017 [39]	Y	Y	Y	Y	N	Y	Y	Y	Y	Y	(+)
Leech, 2016 [40]	Y	Y	Y	Y	N	Y	Y	Y	Y	Y	(+)
Leech, 2017 [41]	Y	Y	Y	Y	N	Y	Y	Y	Y	Y	(+)
Livingstone, 2016 [42]	Y	Y	Y	Y	N	Y	Y	Y	Y	Y	(+)
Livingstone, 2017 [43]	Y	Y	Y	Y	N	Y	Y	Y	Y	Y	(+)
Livingstone, 2018 [44]	Y	Y	Y	Y	N	Y	Y	Y	Y	Y	(+)
Martin, 2017 [45]	Y	Y	Y	Y	N	Y	Y	Y	Y	Y	(+)
Martin, 2019 [46]	Y	Y	Y	Y	N	Y	Y	Y	Y	Y	(+)
Mayr, 2018 [47]	Y	Y	Y	Y	N	Y	Y	Y	Y	Y	(+)
McLeod, 2011 [48]	Y	Y	Y	N	N	Y	Y	Y	Y	Y	(+)
McNaughton, 2008 [49]	Y	Y	Y	Y	N	Y	Y	Y	Y	Y	(+)
McNaughton, 2009 [50]	Y	Y	Y	Y	N	Y	Y	Y	Y	Y	(+)
Milte, 2015 [51]	Y	Y	Y	Y	N	Y	Y	Y	Y	Y	(+)
Milte, 2018 [52]	Y	Y	Y	Y	N	Y	Y	Y	Y	Y	(+)
Morrison, 2012 [53]	Y	Y	Y	Y	N	Y	Y	Y	Y	Y	(+)
Nagle, 2019 [54]	Y	Y	Y	Y	N	Y	Y	Y	Y	Y	(+)
O'Brien, 2014 [55]	Y	Y	Y	Y	Y	Y	Y	Y	Y	Y	(+)
Olstad, 2017 [56]	Y	Y	Y	Y	N	Y	Y	Y	Y	Y	(+)
O'Reilly, 2012 [57]	Y	Y	Y	Y	N	Y	Y	Y	Y	Y	(+)
Petersen, 2015 [58]	Y	Y	Y	N	N	Y	Y	Y	Y	Y	(+)

Quality assessment of studies (ADA Quality Criteria Checklist Primary Research)

Citation	Clear research question?	Free of selection bias?	Study groups comparable?	Method of handling withdrawals described?	Blinding?	Intervention described?	Clear outcomes, valid & reliable measurements?	Appropriate statistical analysis	Conclusions supported by results?	Bias due to funding? Sponsorship unlikely?	(-) or (ϕ) or (+)
Potter, 2014 [59]	Y	Y	Y	Y	N	Y	Y	Y	Y	Y	(+)
Reeves, 2013 [60]	Y	Y	Y	Y	N	Y	Y	Y	Y	Y	(+)
Ribeiro, 2017 [61]	Y	Y	Y	Y	N	Y	Y	Y	Y	Y	(+)
Roach, 2017 [62]	Y	Y	Y	N	N	Y	Y	Y	Y	Y	(+)
Roy, 2016 [63]	Y	Y	Y	N	N	Y	Y	Y	Y	Y	(+)
Roy, 2017 [64]	Y	Y	Y	Y	N	Y	Y	Y	Y	Y	(+)
Russell, 2013 [65]	Y	Y	Y	Y	N	Y	Y	Y	Y	Y	(+)
Russell, 2017 [66]	Y	Y	Y	Y	N	Y	Y	Y	Y	Y	(+)
Shivappa, 2016 [67]	Y	Y	Y	Y	N	Y	Y	Y	Y	Y	(+)
Smith, 2017 [68]	Y	Y	Y	Y	N	Y	Y	Y	Y	Y	(+)
Thorpe, 2013 [69]	Y	Y	Y	Y	N	Y	Y	Y	Y	Y	(+)
Thorpe, 2016 [70]	Y	Y	Y	Y	N	Y	Y	Y	Y	Y	(+)
Vissers, 2016 [71]	Y	Y	Y	Y	N	Y	Y	Y	Y	Y	(+)
Vissers, 2017 [72]	Y	Y	Y	Y	N	Y	Y	Y	Y	Y	(+)
Williams, 2017 [73]	Y	N	Y	N	N	Y	Y	Y	Y	Y	(ϕ)
Wong, 2017 [74]	Y	Y	Y	Y	N	Y	Y	Y	Y	Y	(+)
Wood, 2015 [75]	Y	Y	Y	Y	N	Y	Y	Y	Y	Y	(+)
Zarrin, 2013 [76]	Y	Y	Y	N	N	Y	Y	Y	Y	Y	(+)

Table S2 Overview of components of diet quality indices

	Bread and cereals		Vegetables				Fruits			Red meat, poultry, fish							Protein alternatives			Dairy products							Dairy alternatives			Fats and oils		
	Any	Whole grain	Any	Starchy	Non-starchy	Vegetable variety	Fruit	Fruit juice	Fruit Variety	Meat (not-specified)	Red meat	Poultry	Fish	Other seafood	Lean meat	Ratio of white to red meat	Nuts	Legume	Egg	Milk (not-specified)	Reduced or skimmed milk dairy	Cheese	Yoghurt	Type of milk	Low-fat dairy	Dairy (not-specified)	Soy milk	Dairy alternatives (not-specified)	Core food variety	Fats	Oils MUFA	Oils PUFA
Aust-HEI [77]			Y				Y														Y								Y			
ARFS [13]	Y	Y		Y	Y		Y	Y			Y	Y	Y				Y	Y	Y		Y	Y	Y				Y			Y		
ARFS-1 [15]	Y	Y		Y	Y		Y				Y	Y	Y	Y			Y	Y	Y	Y		Y	Y									
DGI [49]	Y	Y	Y				Y								Y			Y	Y		Y			Y		Y			Y			
Modified DGI [48]	Y		Y			Y	Y		Y	Y																Y						
DGI-2013 [70]	Y	Y	Y				Y								Y		Y	Y	Y		Y	Y	Y	Y		Y		Y	Y	Y	Y	
RDGI [12]	Y	Y	Y				Y				Y		Y							Y	Y	Y		Y								
CSIRO HDS [31]	Y	Y		Y	Y		Y				Y	Y	Y				Y	Y	Y		Y	Y	Y	Y		Y			Y	Y		
TDS [66]	Y	Y		Y	Y	Y	Y				Y		Y		Y						Y	Y	Y			Y						
Aussie-DQI [76]	Y		Y				Y			Y																Y			Y			
HEIFA-2013 [63]	Y	Y		Y	Y	Y	Y		Y		Y			Y	Y			Y			Y			Y	Y					Y	Y	Y

ADQS [21]	Y	Y	Y				Y							Y			Y									Y						
HDHI [74]	Y	Y	Y				Y				Y	Y	Y								Y			Y						Y		
DQI-R [78]	Y		Y				Y																						Y			
RFS [79]		Y		Y	Y		Y	Y				Y	Y		Y		Y	Y	Y		Y	Y	Y		Y							
NRFS [80]	Y										Y																			Y		
MD Score [81]	Y				Y		Y				Y		Y				Y	Y								Y						
MDP index [82]	Y		Y				Y			Y			Y				Y	Y								Y						
MedDiet Score [83]	Y		Y				Y				Y		Y				Y	Y	Y							Y						
MDS [84]	Y		Y				Y			Y			Y	Y			Y	Y								Y						
DASH [85]		Y			Y		Y	Y			Y						Y	Y			Y	Y	Y		Y							
AHEI [86]			Y				Y									Y	Y	Y														
AHEI-2010 [87]		Y	Y				Y	Y			Y						Y	Y														
DQT [57]	Y	Y	Y				Y						Y								Y			Y			Y			Y		
DII [88]																																

Table S2 Overview of components of diet quality indices (continued)

	Discretionary foods												Fluids				Food practice					Other				Macronutrients									Micronutrients		
	Salt	Sugar	Confectionary	Take away food	Meat products	Jam, ice-cream, chocolate	Alcohol	Sugar sweetened beverage	Fried food	Extra food (not specified)	Extras ratio	High saturated fat, low nutrient density food	Water	Tea	Coffee	Other	Low-fat food	Low-salt food	Trimming of fat	Breakfast	Spices	Moderation	Physical activity	Ratio of energy intae to expenditure	Supplement use	Carbohydrate	Total fats	Cholesterol	Saturated fat	MUFA	PUFA	Trans fat	Fats ratio	Protein	Sodium	Mineral	Vitamins
Aust-HEI [77]												Y							Y																		
ARFS [13]						Y	Y																														
ARFS-1 [15]						Y							Y																								
DGI [49]	Y		Y			Y	Y	Y		Y			Y						Y																		
Modified DGI [48]										Y																											
DGI-2013 [illegible]	Y	Y					Y						Y						Y																		
RDGI [12]	Y		Y		Y	Y	Y	Y	Y				Y						Y																		
CSIRO HDS [31]			Y	Y	Y	Y	Y	Y	Y				Y						Y																		
TDS [66]		Y					Y			Y													Y	Y					Y								
Aussie-DQI [76]	Y	Y			Y		Y																						Y								
HEIFA-2013 [63]		Y	Y			Y	Y	Y					Y	Y	Y														Y	Y	Y						
ADQS [21]											Y																						Y	Y			
HDHI [74]	Y			Y				Y	Y								Y	Y	Y	Y																	

DQI-R [78]																					Y					Y	Y	Y							Y	
RFS [79]																																				
NRFS [80]		Y	Y		Y	Y			Y																											
MD Score [81]					Y		Y																													
MDP index [82]					Y		Y																									Y				
MedDiet Score [83]																																Y				
MDS [84]					Y		Y																													
DASH [85]					Y																													Y		
AHEI [86]							Y																	Y	Y						Y	Y				
AHEI-2010 [87]					Y		Y	Y																						Y	Y			Y		
DQT [57]	Y		Y	Y		Y			Y										Y																	
DII [88]							Y							Y	Y						Y					Y	Y	Y	Y	Y	Y	Y		Y		Y	Y

Table S3 Components and scoring of diet quality indices

Australian Healthy Eating Index (Aust-HEI) [77]

Component	Criteria for maximum score	Minimum score*	Maximum score*
Variety	Total number of foods from each food group**(at least once per week)	0 (none)	10
Healthy choices	All healthy choice foods*** (at least once per week)	0 (none)	10
Fruit consumption	≥2 servings per day	0 (none)	10
Vegetable consumption	≥ 4 servings per day	0 (none)	10
Low-fat milk	Low-fat or skim milk	0 (no)	5
Trim fat meat	Usually or do not eat meat	0 (no)	5
High saturated fat, low nutrient density food	Total number of foods eaten (at least once per week or more)	0	10 (none)

*Intermediate amount scored proportionately; **Food groups are vegetables, legumes and fruits; cereals; lean meat and alternatives; dairy; water; *** food items from FFQ such as whole meal bread and cereals, vegetables, fruits and fruit juices, fish and seafood

Australian Recommended Food Score (ARFS) [13]

Food group	Item giving one point	Maximum Score
Vegetable	≥ 4 vegetables per day; Potatoes cooked without fat; Tomato sauce/paste/dried; Tomatoes fresh/canned; Capsicum; Lettuce/endive/salad greens; Cucumber; Celery; Beetroot; Carrots; Cabbage/Brussels sprouts; Cauliflower; Broccoli; Silver beet or spinach; Peas; Green beans; Bean sprouts or alfalfa sprouts; Pumpkin; Onion or leeks; Garlic; Mushrooms; Zucchini	22
Fruit	≥ 2 pieces of fruit/day; ≥1/week of each of Fruit or Vegetable Juice; Canned or frozen Fruit; Oranges or other citrus; Apples; Pears; Bananas; Melons (water, rock, honeydew); Pineapple; Strawberries; Apricots; Peach/nectarines; Mango/pawpaw; Avocado	14
Protein foods	Nuts; Peanut Butter or peanut paste; 1–4/week of each of Beef; Veal; Lamb; Pork; Chicken; Fish, steamed, baked or grilled; Fish, canned (salmon, tuna, sardines); ≥1/ week of each of Baked Beans; Soy beans/soy bean curd/tofu; soya milk; Other beans (chick peas, lentils); use up to 2 eggs per week	14
Grains	≥ 1/week of each of following bread types white high fibre; wholemeal; rye; multigrain; ≥ 4 slices bread per day; ≥1/week AllBran; Sultana bran/FibrePlus/ Branflakes; WeetBix/ VitaBrits/ Weeties; Rice; Pasta/noodles Vegemite/marmite/promite; Cornflakes/ Nutragrain/ SpecialK; Porridge; Muesli	14
Dairy	Reduced fat or skim; > 500mL/day; Cheese ≤1/week; Ice-cream; Yoghurt ≥1/week; use ricotta/cottage cheese; use low fat cheese	7
Fats	Use nil/polyunsaturated/ monounsaturated margarine	1
Alcohol	Drink beer/wine/spirits -up to 4 days/week; 1 or 2 glasses maximum/day	2

Australian Recommended Food Score-1 (ARFS-1) [15]

Food group	Item giving one point	Maximum Score
Vegetables	3-4 nightly meals with vegetables; ≥1 per day of each of the following vegetables: Potatoes, Pumpkin, sweet potato, Cauliflower, green beans,	21

	spinach, Cabbage or Brussels sprouts, peas, Broccoli, Carrots, Zucchini or eggplant or squash, Capsicum, corn, Mushrooms, Tomatoes, Lettuce, Celery or Cucumber, avocado, Onion or leeks or shallots/spring onion	
Fruit	≥ 1 pieces of fruit/day; ≥1/week of each of the following Fruit: Canned fruit, fruit salad, dried fruit, Apples or Pears, orange or mandarin or grapefruit, Banana, Peach or nectarines or plum or apricot, Mango or pawpaw, Pineapple, grapes or Strawberries or blueberries, Melons (any variety)	12
Protein foods-Meat/flesh	≤ 1 serve of minced meat per month but greater than never; 1-4 serves per week of: beef or lamb with or without sauce and or vegetables per week chicken without batter or crumbing but with or without sauce and/or vegetables; ≥1 per week of fresh fish, canned tuna or salmon or sardines, other seafood (eg, prawns, lobster)	7
Vegetable sources of protein	≥1 per week of following: nuts (eg, peanuts, almonds), nut butters, eggs, soybeans or tofu, baked beans, other beans or lentils (eg, chickpeas, split peas)	6
Breads and cereals	Usual bread choice is "other" (eg, rye, high fibre white); ≥ 1/week of each of following: Muesli, cooked Porridge, breakfast cereal (eg, WeetBix/ Nutrigrain/ Cornflakes), bread or pita bread or toast, english muffin or bagel or crumpet, rice, other grains (eg, couscous, burghul), noodles (eg, egg noodles, rice noodles), pasta, tacos or burritos or enchiladas, clear soup with rice or noodles	13
Dairy	≥ 2 serves of milk, yogurt or cheese per day; ≥ 1 serve per week but ≤1 serve per day of flavoured milk, ice-cream, frozen yogurt; ≥ 1 serve per week but ≤4 serves per day of Cheese, cheese spread or cream cheese; ≥1 serve per week of plain milk, yogurt (not frozen), cottage cheese or ricotta	11
Water	≥ 4 glasses of water (including tap, unflavoured bottled water and unflavoured mineral water)	1
Spreads/sauces	≥ 1 serve per week of; yeast extract spread; tomato or barbecue sauce	2

Dietary Guideline Index (DGI) [49]

Dietary Guideline	Detailed description	Criteria for minimum score (0)	Criteria for maximum score (10) *
Dietary variety **	Proportion of foods for each core food group that are consumed at least once per week**	0%	100%
Fruit	Servings per day	0	≥ 2
Vegetables and legumes	Servings per day	0	≥ 5
Cereals	Frequency of consumption of breads and cereals per day	0	19–60 y: M ≥ 6, F ≥ 4; 60 y: M ≥ 4, F ≥ 4
Whole grain cereals	Proportion of whole-meal/whole-grain bread consumed relative to total bread	0%	100%
Meat and alternatives,	Frequency of consumption of lean meats and alternatives per day	0	≥ 1
Lean protein sources	Proportion of lean meats and alternative relative to total meats and alternatives	0%	100%
Dairy products	Frequency of consumption of dairy products per day	0	≥ 2

Low-fat/ reduced fat dairy	Type of milk usually consumed	Whole milk	Low-fat milk
Fluids	Frequency of consumption of beverages[1]	0	≥ 8
	Proportion of water consumed relative to total beverages[1***]	0%	50%
Saturated fat intake	Type of milk usually consumed[1]	Whole milk	Low-fat milk
	Trimming of fat from meat[1]	Never or rarely	Usually
Salt use	In cooking[1]	Usually	Never or rarely
	At the table[1]	Usually	Never or rarely
Alcoholic beverages	Frequency of consumption of all alcoholic beverages per day	M≥ 4, F≥ 2	M ≥ 2, F ≥ 1
Added sugar	Frequency of consumption of soft drink, cordial, fruit juice drink, jam, chocolate, confectionary per day	19–60 y: M >1.5, F > 1.25;<br>>60 y: M >1.25, F > 1	19–60 y: M <1.5, F < 1.25;<br>>60 y: M <1.25, F < 1
Extra food	Frequency of consumption of extra foods per day	19–60 y: M >3, F > 2.5;<br>>60 y: M >2.5, F > 2	19–60 y: M <3, F < 2.5;<br>>60 y: M <2.5, F < 2

[1]DGI sub-components ranged from 0-5; *Intermediate amount scored proportionately except extra food, saturated fat, salt, sugar and alcohol.; **Variety within each core food group (fruits, vegetables, meat/protein, dairy and cereals) was calculated [core foods consumed at least once per week divided by total number of core foods listed in FFQ], assigned a score of 2 and total score was obtained by the sum of each group; *** Proportion of water to total beverage intake was derived from US beverage guidelines [89].

Modified Dietary Guideline Index (Modified DGI) [48]

Component	Description	Minimum score (0)*	Maximum score (10)
Breads and cereals	Servings per day	0	≥ 4
Vegetables	Servings per day	0	≥ 5
Fruit	Servings per day	0	≥ 2
Dairy	Servings per day	0	≥ 2
Meat and alternatives	Servings per day	0	≥ 1
Extra foods	Servings per day	>2.5	≤ 2.5
Vegetable variety	Proportion of vegetables listed in FFQ consumed in last 12 months	0%	100%
Fruit variety	Proportion of fruits listed in FFQ consumed in last 12 months	0%	100%

*Intakes between minimum and maximum scored proportionately

Dietary Guideline Index-2013 (DGI-2013) [70]

2013 Dietary Guideline	Detailed description	Criteria for minimum score*	Criteria for maximum score*	Maximum score*
Guidelines for adequate intake				
1. Enjoy a wide variety of nutritious foods	Food variety: Proportion of foods for each core food group that are consumed at least one serve per week	0%	100%	10
2. Plenty of vegetables	Total vegetable intakes; Servings of vegetables per day	0	19-50 y: M≥6, F≥5<br>51-70 y: M≥5.5, F≥5<br>>70 y: M≥5, F≥5	10
3. Fruit	Total fruit intakes; Servings of fruit per day	0	≥ 2	10

4. Grain (cereal) foods	Total cereal intake: Servings of grains per day	0	19-50 y: M≥6, F≥6<br>51-70 y: M≥6, F≥4<br>>70 y: M≥4.5, F≥3	5
	Mostly whole grain or high fibre cereals: type of bread usually consumed	White bread	Wholemeal bread	5
5. Lean meat and poultry, fish, eggs, nuts and seeds, and legumes/ beans	Total meat and alternative: servings per day	0	19-50 y: M≥3, F≥2.5<br>51-70 y: M≥2.5, F≥2<br>>70 y: M≥2.5, F≥2	5
	Lean meat: Proportion of lean meats and alternatives to total meats and alternatives per day	0%	100%	5
6. Milk, yoghurt, cheese and/or their alternatives	Total dairy and alternative: servings per day	0	19-50 y: M≥2.5, F≥2.5<br>51-70 y: M≥2.5, F≥4<br>>70 y: M≥3.5, F≥4	10
7. Drink plenty of water	Total beverage intake: servings per day	0	M ≥10; F ≥8	5
	Water: proportion of water to total beverage intake per day	0%	≥50%	5
Guidelines to limit or moderate intakes				
8. Limit intake of foods containing saturated fat, added salt, added sugar and alcohol	Limit discretionary foods	M >3; F>2.5	M≤3, F≤2.5	10
9. Limit intake of foods high in saturated fat	Trim meat; trimming fat from meat	Never or rarely	Usually	5
	Choose reduced-fat milk: type of milk usually consumed	Whole milk	Skimmed, low or reduced fat milk	5
10. Small allowance of unsaturated oils, fats or spreads	Unsaturated spreads and oils: servings per day	M >4; F>2	19-50 y: M≤4, F≤2<br>51-70 y: M≤4, F≤2<br>>70 y: M≤2, F≤2	10
11. Limit intake of foods and drinks containing added salt	Salt use: salt added during cooking	Usually	Never or rarely	5
	Salt use: salt added during the meal	Usually	Never or rarely	5
12. Limit intake of foods and drinks containing added sugar	Limit extra sugar: servings per day	M >1.5; F>1.25	M ≤1.5; F≤1.25	10
13. Limit alcoholic intakes if chosen to drink	Limit alcohol: servings per day	>2	≤ 2	10

*Intermediate amount scored proportionately.

RESIDential Environments (RESIDE) Dietary Guideline Index (RDGI) [12]

ADG Component	RESIDE Indicator Survey Item	Criteria for minimum	Criteria for intermediate	Criteria for maximum
Vegetables (fresh, frozen and tinned)	Eating vegetables each day in serves	Do not eat=0	19-70 y M: ≤1 serve =2, 2 serves=4, 3-4 serves=6, 5 serves=8<br>F and >70 y M: ≤1 serve =2.5, 2 serves=5, 3-4 serves=7.5	19-70 y M: 6 serves=10<br><br>F and >70 y M: ≥5 serves=10
Fruit (fresh, frozen and tinned)	Eating fruit each day in serves	Do not eat=0	≤1 serve =5	≥2 serves= 10

Grains/cereals: mostly wholegrain and/or high cereal fibre varieties	Type of bread	White bread=0	Don’t eat bread/other=1.25	High fibre white, wholemeal, multigrain, rye, spelt=2.5
	Frequency of eating bread (bread rolls, flat breads, crumpets, bagels, English or bread type muffins)	< once per month=0	Once per month=0.5<br>2-3 times per month=1<br>1-2 times per week=1.5<br>3-5 times per week=2	6-7 times per week=2.5
	Frequency of eating pasta, rice, noodles or other cooked cereals?	< once per month=0	Once per month=1<br>2-3 times per month=2<br>1-2 times per week=3<br>3-5 times per week=4	6-7 times per week=5
Lean meats	Frequency of eating red meat (beef, lamb, and kidney but not pork or ham; chops, steaks, roast, rissoles, mince, stir-fries and casseroles)[1]	6-7 times per week=0	3-5 times per week=2.5	≤1-2 times per week=5<br>Don’t eat meat=5
	Frequency of eating fish[2]	< once per month=0	Once per month=1.25<br>2-3 times per month=2.5<br>1-2 times per week=3.75	≥3-5 times per week =5
Dairy or alternatives: mostly reduced fat	Total amount of milk consumed a day	<150 ml=0	>70 y, 51-70 F: 150-600 ml=1.25<br>19-50 y, 51-70 M: 150-300 ml=1.25	>70 y, 51-70 F: >600 ml=2.5<br>19-50 y, 51-70 M: ≥301 ml=2.5
	Type of milk	Whole (full cream)= 0	Low or reduced fat/other=1.25	Skim=2.5
	Eating cheese	< once per month=0	Once per month=1<br>2-3 times per month=2<br>1-2 times per week=3<br>3-5 times per week=4	6-7 times per week =5
Drinking plenty of water	Cups of water<br>Cups of diet or sugar-free soft drinks, cordial or sports drink in a day (coke zero or sugar free Gatorade)<br>Cups of hot drinks (tea, coffee, herbal tea)	Total beverage intake zero cups=0	Total beverage intake<br>M 1-9 cups=2.5<br>F 1-7 cups=2.5	Total beverage intake<br>M ≥10 cups=5<br>F ≥8 cups=5
	Proportion of water to total beverage intake[3]	0%=0	>0% <50%=2.5	≥50%=5
Limit intake of food high in saturated fat	Frequency of eating chips, French fries, wedges, fried potatoes or crips[4]	6-7 times per week=0	3-5 times per week=0.5<br>1-2 times per week=1<br>2-3 times per month=1.5	≤ once per month=2

	Frequency of eating meat products such as sausages, frankfurters, polony, meat pies, bacon or ham[4]	6-7 times per week=0	3-5 times per week=0.5<br>1-2 times per week=1<br>2-3 times per month=1.5	≤ once per month=2
	Frequency of eating trimmed of fat either before or after cooking[4]	Never or rarely=0	Sometimes=1	Usually=2
	Frequency of eating fried, roast or BBQ chicken, pizza, burgers or fish and chips[4]	6-7 times per week=0	3-5 times per week=0.5<br>1-2 times per week=1<br>2-3 times per month=1.5	≤ once per month=2
	Frequency of eating meat pies, sausage rolls or other savoury pastries[4]	6-7 times per week=0	3-5 times per week=0.5<br>1-2 times per week=1<br>2-3 times per month=1.5	≤ once per month=2
Limit intake of food and drinks containing added salt	Adding salt after cooking	Usually=0	Sometimes=2.5	Never or rarely=5
	Adding salt during cooking	Usually=0	Sometimes=2.5	Never or rarely=5
Limit intake of food and drinks containing added sugar	Eating biscuits, cakes, desserts, pastries, lollies and/or chocolate[4]	6-7 times per week=0	3-5 times per week=0.5<br>1-2 times per week=1<br>2-3 times per month=1.5	≤ once per month=5
	Drinking regular or sugar sweetened soft drinks, cordial, fruit juice or sport drinks[5]	>2 cups=0	1.5-2 cups=2.5	≤1 cup=5<br>No response=5
Drinking alcohol	Days of drinking alcohol	≥6 days per week=0	2-5 days per week=2.5	≤ once per week=5<br>Don't drink alcohol=5
	Standard drinks on a day if alcohol is consumed	>4 drinks=0	3-4 drinks=2.5	≤2 drinks=5

*Intermediate amount scored proportionately; [1]Australian adults eat meat in larger portion sizes than standard sizes, thus consumption of red meat 6-7 times/week was considered greater than recommendation. (The maximum recommendation for lean, cooked red meat is 455g (7 serves)/week); [2]The Australian Heart Foundation recommends eating fish at least 2-3 times/week; [3] Nutrient Reference Values for Australia and New Zealand were used. Total beverage excludes alcohol and sugar sweetened drinks. Proportion of water to total beverage intake was based on methods of McNaughton et al (2008) and Thorpe et al (2016) based on US beverage guidelines; [4]The ADG's guideline recommendation for discretionary foods for taller or more active are 2.5 serves in women and 3 serves in men. Maximum points were given to the lowest intakes with proportionate scores for intakes above that; [5]The American Heart Association recommends about added sugar; men 1.5 serves or 9 teaspoons/d and women 1.25 serves or 6 teaspoons/d

Commonwealth Scientific and Industrial Research Organization Healthy Diet Score (CSIRO HDS) [31]

Component	Criteria for minimum	Criteria for maximum		Score range
		Male	Female	
Fruit	0 serving	> 2 servings	>2 servings	0-10
Vegetables (starchy, salad, cooked)	0 serving	6 servings	5 servings	0-10

Bread and cereals	0 serving	6 servings	6 servings	0-5
Meat and alternatives (red meat, poultry, fish, eggs, legumes, tofu, nuts, seeds or other meat alternatives)	0 serving	3 servings	2 1/2 servings	0-10
Dairy and dairy substitute (milk, cheese, yoghurt)	0 serving	2 1/2 servings	2 1/2 servings	0-5
Fluids (water, fruit juice, soft drink, cordial or sports drink)	0% water	100 % water	100 % water	0-10
Discretionary food<br>-times of takeaway food consumption<br>-times of eating snack type bar<br>-servings of processed meat<br>-servings of potato chips<br>-servings of savoury snacks (crips, pretzels or crackers)<br>-servings of pies or savoury pastries<br>-servings of sweet biscuits/ cakes/ buns/ muffins/ doughnuts<br>-servings of chocolate or lollies<br>-servings of ice-cream or ice-blocks<br>-servings of alcohol drinks	Number of times: ≥ 2	≤3 servings	≤2 1/2 servings	0-20
Food quality (whole grain)	Never	Always	Always	0-5
Food quality (milk)	Whole fat	Skim (< 1%)	Skim (<1%)	0-5
Healthy fats (spreads)	Butter	Unsaturated margarine	Unsaturated margarine	0-5
Healthy fats (trimmed meat)	Never	Always	Always	0-5
Variety (types of fruits in 2 days)	0	≥ 4	≥ 4	0-2
Variety (types of vegetables in 2 days)	0	≥ 5	≥ 5	0-2
Variety (types of dairy foods in 2 days)	0	≥ 5	≥ 5	0-2
Variety (types of protein-based foods in 2 days)	0	≥ 13	≥ 13	0-2
Variety (types of grain-based foods in 2 days)	0	≥ 9	≥ 9	0-2

*Intermediate amount scored proportionately.

Total Diet Score (TDS) [66]

Component/ Dietary Guideline	Criteria for scoring	Component sub-score		Score range
Eat plenty of vegetables, legumes and fruit	Total vegetable serves/day	7 serves<br>5.6 serves<br>4.2 serves<br>2.8 serves<br>1.4 serves	0.5<br>0.4<br>0.3<br>0.2<br>0.1	0-2
	Vegetable variety score/day	≥1 serve green<br>≥ 1 serve orange<br>≥ 1 serve of cruciferous<br>≥ 1 serve of tuber or bulb<br>≥ 0.5 serve of legumes	0.1<br>0.1<br>0.1<br>0.1<br>0.1	
	Total fruit serves/day	3 serves<br>2 serves	1<br>0.5	
Eat plenty of cereals, preferably whole grain/ meal	Total cereals serves/d<br>Women	4 serves<br>3 serves<br>2 serves	1<br>0.75<br>0.5	0-2

	Men<br><br><br><br><br><br>Whole-grain cereal serves/d<br>Women<br><br><br><br>Men	1 serve<br><br>6 serves<br>5 serves<br>4 serves<br>3 serves<br>2 serves<br>1 serve<br><br>4 serves<br>3 serves<br>2 serves<br>1 serve<br><br>6 serves<br>5 serves<br>4 serves<br>3 serves<br>2 serves<br>1 serve	0.25<br><br>1<br>0.83<br>0.66<br>0.5<br>0.33<br>0.166<br><br>1<br>0.75<br>0.5<br>0.25<br><br>1<br>0.83<br>0.66<br>0.5<br>0.33<br>0.166	
Include lean meats, fish, poultry and/or alternatives	Meat/ alternative/ day<br>Lean red meat/ week (i.e. 0.428/day)	≥1 serve<br>≥3 serves	1.5<br>0.5	0-2
Include milk, yoghurts, cheese and/or alternatives	Total dairy serves/d<br><br><br><br><br><br>Ratio of skimmed/low fat (S/LF) intake: whole milk intake	≥2–3 serves<br>≥3–4 serves<br>1–<2 serves<br>>4 serves<br>0–<1 serves<br><br>S/LF>whole milk<br>S/LF=whole milk<br>Whole milk>S/LF	1.5<br>1<br>1<br>0.5<br>0<br><br>0.5<br>0.25<br>0	0-2
Limit saturated fat and moderate total fat intake	Percentage of energy (% E) from saturated fat<br><br><br>Fish serves/week	<10% E<br>10–12% E<br>>12% E<br><br>≥ 2 serves<br>1–<2 serves<br><1 serve	1<br>0.5<br>0<br><br>1<br>0.5<br>0	0-2
Choose foods low in salt	Na intake/d	≤ 40 mmol (920 mg)<br>> 40–≤100 mmol (920–2300 mg)<br>>100 mmol (2300 mg)	2<br>1<br>0	0-2
Limit alcohol intake if you choose to drink	Alcohol intake/d<br>Women<br><br><br><br>Men	 ≥0 g–<10 g<br>≥10 g–<20 g<br>≥20 g<br><br>≥0g–<20 g<br>≥20 g	 2<br>1<br>0<br><br>2<br>0	0-2
Consume only moderate amounts of sugars and foods with added sugars	Percentage of energy from sugar	<15% total energy<br>≥15–<20% total energy<br>≥20% energy	2<br>1<br>0	0-2
Extra foods, not essential to provide nutrients and maybe high in salt, fat or sugar	Extra food serves/d<br>Women	 <2·5 serves<br>2·5–<4 serves<br>>4 serves	 2<br>1<br>0	 0-2

	Men	<3 serves/d<br>3–<5 serves<br>≥5 serves	2<br>1<br>0	
Prevent weight gain: be physically active and eat according to energy needs	Ratio of energy intake to energy expenditure<br><br>Physical activity (METs)	0·76–1·24<br><0·76 or >1·24<br><br>Lowest tertile<br>Middle tertile<br>Highest tertile	1<br>0<br><br>0<br>0.5<br>1	0-2

Aussie-Diet Quality Index (Aussie-DQI) [76]

Component	Criteria for minimum score	Criteria for maximum score	Score range**
Vegetables	0 serving/day	≥5 servings/day	0-10
Fruits	0 serving/day	≥2 servings/day	0-10
Dairy products	0 or >4 servings/day	2-4 servings/day	0-10
Meat and alternatives	0 from meat and alternative/day or > 2 servings from meat/day	½ to 1½servings per day	0-10
Cereals	19–60 y: M 0 or >12 servings/day, F 0 or > 9 servings/day;<br>60 y: M 0 or > 9 servings/day, F 0 or >7 servings/day	19–60 y: M 6-12 servings/day, F 4-9 servings/day;<br>60 y: M 4-9 servings/day, F 4-7 servings/day	0-10
Percentage of total energy from saturated fatty acid (SFA)*	>10% of total energy	≤10% of total energy	0-10
Percentage of total energy from sugar*	>15% of total energy	≤ 15% of total energy	0-10
Alcohol	M >40 g/day<br>F >20 g/day	M 0-20 g/day<br>F 0-10 g/day	0-10
Processed meat*	>5 servings/month	0-5 servings/ month	0-10
Added salt/ sodium	>2300 mg/day sodium or > 6 g/day salt	≤2300 mg/day sodium or ≤6 g/day salt	0-10
Variety§<br>Vegetables<br>Fruits<br>Whole grain<br>Fish	 0 serving/day<br>0 serving/day<br>0 serving/day<br>0 serving/day	 ≥ 3 types of vegetables/day<br>≥ 2 types of fruits/day<br>Whole grain cereal/day<br>Fish in daily or usual diet	 0-5<br>0-5<br>0-5<br>0-5

** Proportional scoring were assigned for scores between minimum and maximum; * No clear cut-offs were defined for the intakes of SFA, sugar in Australian dietary guidelines. Other relevant dietary guidelines (WHO, US, UK), national consumption level and findings from similar studies were applied here; § For calculation of variety, ***vegetables*** were subdivided into 3 subgroups (group A: a score of 2 was assigned for any legume intake; group B: score of 1.5 was assigned to any consumption of tomato, carrot or fruiting vegetables; group C: score of 1.5 was assigned to any type of dark leafy green vegetables): ***fruits*** were subdivided into 3 subgroups (group A: berries; group B: citrus and tropical fruits; group C: dried, stone and other fruits) and score of 5 was assigned for consumption of any fruit groups: score of 5 was assigned for inclusion of any ***wholegrain cereals*** and score of 5 was assigned for inclusion of any ***fish products***

Healthy Eating Index for Australian Adults-2013 (HEIFA-2013) [63]

Component	Criteria for minimum score	Criteria for maximum score	Intermediate scores**

Frequency of consumption of extra foods per day e.g., 1 slice (40 g) plain cake or small cake-type muffin	Discretionary foods serves /d:<br>M: ≥6= 0,<br>F: ≥5.5= 0	Discretionary foods serves/d:<br>M: <3 = 10<br>F: <2.5= 10	Discretionary foods serves/d:<br>M: 3-3.9 = 7.5<br>4-4.9= 5<br>5-5.9= 2.5<br>F: 2.5-3.4= 7.5<br>3.5-4.4= 5<br>4.5-5.4= 2.5
Total vegetables: servings of vegetables and legumes per day e.g., 1 (75 g) medium tomato Variety of vegetable servings/d	Total vegetables 0<br><br>Variety 0	M: ≥6 serves<br>F: ≥5 serves<br><br>Varied vegetables e.g., green, orange, cruciferous, tuber, or bulb and legumes consumed	Total vegetable serves/ d<br>M: ≥ 6= 5<br>4.8-5.9= 4<br>3.6-4.7= 3<br>2.4-3.5= 2<br>1.0-2.3= 1<br>No vegetable= 0<br>F: ≥ 5= 5<br>4.0-4.9= 4<br>3.0-3.9= 3<br>2.0-2.9= 2<br>1.0-1.9= 1<br>No vegetable= 0<br><br>Vegetable variety score/d<br>≥ 1 serve green= 1<br>≥ 1 serve orange= 1<br>≥ 1 serve cruciferous= 1<br>≥ 1 serve tuber/bulb= 1<br>≥ 0.5 serve legume= 1
Fruit servings per day; variety of fruit servings/d e.g., 1 (150 g) medium apple, banana, orange, or pear	No fruit, whole fruits, 0<br><br>Variety 0	Fruit serves/d:<br>≥2 = 5<br><br>Varied fruits consumed	Fruit serves/d:<br>1.5-1.9 = 3.75<br>1.0-1.4 = 2.50<br>0.5-0.9 = 1.25<br>0-0.4=0<br><br>Fruit variety score/d<br>≥ 2 varieties= 5
Whole grains/ Refined grains/ Cereals: frequency of consumption of breads and cereals per day.<br>Whole-grain cereals: proportion of whole meal/whole-grain bread consumed relative to total bread e.g., 1 slice (40 g) bread	<50% whole grains<br>M: ≤ 6, F: ≤6	Whole grains: 50% whole grains<br>M: ≥6, F: ≥6	Total cereal serves/d:<br>6 = 5<br>5 = 4.17<br>4 = 3.34<br>3 = 2.5<br>2 = 1.67<br>1 = 0.84<br><br>Whole-grain cereal serve/d<br>≥ 3 = 5<br>2.5-2.9= 4<br>2.0-2.4= 3<br>1.5-1.9= 2<br>1.0-1.4= 1<br>No whole-grain= 0
Total protein foods, seafood and plant proteins, and lean meat and meat alternatives: frequency of consumption of lean meats and alternatives per day e.g., 65	No protein foods	M: ≥3= 10<br>F: ≥ 2.5= 10	Meat/ meat alternative serves/ d<br>M: 2.5-2.9= 8<br>2.0-2.4= 6

g cooked lean red meat such as beef, lamb. One cup (150 g) cooked or canned legumes/beans	No seafood or plant proteins 0		1.5-1.9= 4<br>1.0-1.4= 2<br>0.5-0.9= 1<br>≤0.5= 0<br>F: 2.0-2.4= 8<br>1.5-1.9= 6<br>1.0-1.4= 4<br>0.5-0.9= 2<br>≤0.5= 0
Low-fat dairy consumption/ dairy foods/ alternatives: frequency of consumption of dairy products per day<br>Low-fat/reduced-fat dairy: type of milk usually consumed e.g., 1 cup (250 mL) fresh milk	No dairy	M: ≥2.5, F: ≥2.5= 10	Dairy/ dairy alternatives serves /d<br>2.0-2.4= 8<br>1.5-1.9= 6<br>1.0-1.4= 4<br>0.5-0.9= 2<br>No =0
Fluids: proportion of water consumed relative to total beverages<br>Includes water/tea/coffee[¥]	No water= 0	≥50% water consumed relative to total beverages = 5	Water consumed/ total beverages per day<br>40-49%= 4<br>30-39%= 3<br>20-29%= 2<br>10-19%= 1
Consumption of high saturated fat, nutrient low-density foods, and fatty acids e.g., PUFA and MUFA: 10 g margarine/7 mL oil	% energy intake (% E): saturated fat >10%,<br><br>PUFA and MUFA<br>M: 1 serve<br>F: < 0.5 serve	% energy intake (% E): total saturated fat <10%,<br><br>PUFA and MUFA<br>M: 4 serves<br>F: 2 serves	Saturated fat<br>≤ 10% E = 5<br>10.1-12% E = 2.5<br>> 12% E = 0<br><br>PUFA & MUFA serves/ d<br>M: 4 = 5<br>3-3.9= 3.75<br>2-2.9= 2.5<br>1-1.9= 1.25<br>0-0.9= 0<br>F: 2 = 5<br>1.5-1.9= 3.75<br>1-1.4 = 2.5<br>0.5-0.9= 1.25<br>0-0.4 = 0
Sodium options, salt used in cooking and at the table	≥100 mmol (2300 mg) of Na/d = 0	0-70 mmol (920-1610 mg) Na/d= 10	Sodium/ d<br>70-99 mmol (1610-2300 mg)= 5
Consume only moderate amounts of sugars and foods containing added sugars.<br>Added sugars: frequency of consumption of soft drink, cordial, fruit juice drink, jam, chocolate, confectionary, grain desserts (e.g., cakes, pies), dairy desserts, and candy per day.<br>Percentage of energy (% E) from sugar[§]	>1.5 serves<br>>20% E= 0	<1.5 serves<br><15% E=10	Food containing added sugar/ d<br>15-19% E= 5
Alcohol: frequency of consumption of all alcoholic beverages per day 200 mL wine (2 standard drinks)	>2 per day Only scored out of 5	<2.0 per day	≤ 2 per day= 5

[¥] No quantitative Australian guideline, scoring from DGI was used; [§] Since there was no quantitative guidelines for added sugar, one-half of the discretionary foods guidelines was used;***Composite subscores were calculated based on factors derived from the serving recommendations given in AGHE

Australian Diet Quality Score (ADQS) [21]

Food group	RDI (-10%)	Mean intake (g or kJ)/day (-10%)	Scoring
Vegetables	Men= 371.25 g<br>Women=337.5g<br>Average RDI used=350g	118.20g	1 point=0-118.2<br>2 points=118.2-350<br>3 points=350+
Fruit	270 g	158.60 g	1 point=0-158.6<br>2 points=158.6-270<br>3 points=270+
Whole grains	1500kJ	733.95kJ	1 point=0-733.95<br>2 points=733.95-1500<br>0 point=1500+
Processed grains	1500kJ	815.57kJ	1 point=0-815.57<br>2 points=815.57-1500<br>0 point=1500+
Dairy	1250kJ	752.62kJ	1 point=0-752.62<br>2 points=752.62-1250<br>1 point=1250+
Protein	Men= 1250-1500kJ<br>Women=1000-1250 kJ<br>Average RDI used=1250kJ	804.13kJ	1 point=0-804.13<br>2 points=804.13-1250<br>1 point=1250+
Nuts	30g	9.68g	1 point=0-9.68<br>2 points=9.68-30<br>3 points=30+
Seafood	100g	41.35g	1 point=0-41.35<br>2 points=41.35-100<br>3 points=100+
Fats ratio	Proportion of Unsaturated Fats to Saturated Fats		0 point=0-0.3<br>1 point=0.3-0.6<br>2 points=0.6-1.0
Extras ratio	Proportion of Total Daily kJ received from Extras		2 points=0-0.25<br>1 point=0.25-0.5<br>0 point=0.5-1.0

Based on mean intake and Recommended Daily Intake (RDI)

Healthy Dietary Habits Index (HDHI) [74]

Item	Description	Scoring criteria*				
		0	1	2	3	4
Red meat	Trimming meat fat before consumption	Never	Rarely	Sometimes	Regularly	Always
Chicken	Trimming chicken fat before consumption	Never	Rarely	Sometimes	Regularly	Always
Fish/shellfish	Proportion of fried fish/shellfish relative to total fish/shellfish intake	Never consume fish/ shellfish	76-100%	51-75%	26-50%	0-25%
Milk	Types of milk consumed	None	Whole or standard milk	Other (rice, goat milk)	Reduced fat/ soy milk	Skim or trim milk
Spread	Types of fat spread used	Butter	Butter and margarine blend	Margarine** (full fat)	Plant sterol margarine***	None/ margarine (light or reduced fat)
Low-fat foods	Use of low-fat products	Never	Rarely	Sometimes	Regularly	Always

Fries	Intake of potatoes and kumara (sweet potato) fries per week	≥7 times/ week	5-6 times/ week	3-4 times/ week	1-2 times/ week	Never, < 1 time/week
Bread	Types of bread consumed	Don't eat bread	White/other		§ Light grain/ high fibre white bread	Heavy grain bread§§
Fruit	Fruit intake per day	Never	<1 serving	1 serving		2-4 servings
Vegetables	Vegetables intake per day	Never	< 1 serving	1 serving	2 servings	≥3 servings
Soft drink	Soft drink or energy drink consumption per week	≥7 times/ week	5-6 times/ week	3-4 times/ week	1-2 times/ week	Never, < 1 time/week
Breakfast	Breakfast consumption per week	0	1-2	3-4	5-6	7
Fast foods	Purchasing fast food or takeaways	≥7 times/ week	5-6 times/ week	3-4 times/ week	1-2 times/ week	Never, < 1 time/week
Added salt	Adding salt to foods before eating	Always	Regularly	Sometimes	Rarely	Never
Low salt food	Use of low-salt products	Never	Rarely	Sometimes	Regularly	Always

* Scoring was based on responses from Dietary Health Questionnaire (DHQ); ** Fat spread made from vegetable oils such as canola, sunflower and olive oils; *** Margarine spread containing phytosterols, both full and low-fat varieties such as Proactive and Logicol; § Including commercial brands such as Molenberg, Freya's, Ploughmans, and MacKenzie High Country; §§ Including commercial brands such as Vogels and Burgens

Diet Quality Index-Revised (DQI-R) [78]

Component	Score	Scoring criteria
Total fat ≤30% energy intake	0-10 points	≤30% = 10<br>>30, ≤40= 5<br>>40=0
Saturated fat ≤10% energy intake	0-10 points	≤10%=10<br>>10, ≤13=5<br>>13%=0
Dietary cholesterol <300 mg/day	0-10 points	≤300mg=10<br>>300, ≤400 mg=5<br>>400 mg=0
2-4 servings fruit per day, % recommended servings	0-10 points	Calculated as continuous variable.
3-5 servings vegetables per day, % recommended servings	0-10 points	Calculated as continuous variable.
6-11 servings grains per day, % recommended servings	0-10 points	Calculated as continuous variable.
Calcium intake as % AI for age, % recommended servings	0-10 points	Calculated as continuous variable.
Iron intake as % 1989 RDA for age	0-10 points	Calculated as continuous variable.
Diet diversity score *<br>• Grains (7 items)<br>• Vegetables (7 items)<br>• Fruits (3 items)<br>• Meat and dairy (7 items)	0-10 points<br>(2.5 points in each subscale)	≥6 =10<br>≥3, <6 =5<br><3 =0
Dietary moderation score**	0-10 points	≥7 =10

• Added sugar<br>• Discretionary fat<br>• Sodium<br>• Alcohol	(2.5 points in each subscale)	≥4, <7 =5<br><4 =0

* Each item in variety and moderation scored 1 point if ≥ ¼ of daily serve consumed and zero point otherwise. All points are summed across item and divided by total number of items in that group, then multiplied by 2.5 (because the top score is 2.5 points for each group). The highest score 10 points are assigned for diet diversity if total points for all 4 groups ≥6, 5 points if between 3 to 5.9, and zero if <3; ** The highest score 10 points are assigned for moderation if total points for all 4 groups ≥7, 5 points if between 4 to 6.9, and zero if <4.

Recommended Food Score (RFS) [79]

Components	Criteria for scoring*
1. Fruit<br>1.a Apples or pears<br>1.b Oranges<br>1.c Cantaloupe<br>1.d Orange or grapefruit juice<br>1.e Grape fruit<br>1.f Other fruit juice	Intake above the following cut points were assigned a score of 1.<br>15g/day for non-beverages<br>30g/day for beverages<br>For "RFS-Median", sex-specific median intakes were used as cut points, and intakes above the median were assigned a score of 1.<br>This scoring was used with 24-h recall data.
2. Vegetables<br>2. a Dried beans<br>2. b Tomatoes<br>2. c Broccoli<br>2. d Spinach<br>2. e Mustard, turnip, or collard greens<br>2. f Carrots or mixed vegetables with carrots<br>2. g Green salad<br>2. h Sweet potatoes, yam<br>2. i Other potatoes	
3. Whole grains<br>3. a Dark breads like wholewheat, rye or pumpernickel<br>3. b Corn bread, tortillas, and grits<br>3. c High fibre cereals such as bran, granola or shredded wheat<br>3. d Cooked cereals	
4. Lean meats and alternatives<br>4. a Baked or stewed chicken or turkey<br>4. b Baked or broiled fish	
5. Low-fat dairy<br>5. a 2% milk and beverages with 2% milk<br>5. b 1% or skimmed milk	

* Another variant of scoring was assigning a score of 1 for each recommended food consumed more than once per week. A total of 49 foods (fruit, vegetable, whole grain, lean meat and alternatives, low-fat dairy) listed in FFQ were included.

Not Recommended Food Score (NRFS) [80]

Component	Criteria for minimum score of 0	Criteria for maximum score of 1
Meat and its product-9 points<br>Meat; Meat stew; Minced meat; Bacon; Sausages; Blood pudding/ sausages; Cold cuts; Pate; Liver/ kidney	Never/ seldom	At least 1-3 times per month
Fried food- 3 points<br>Fried potatoes; French fries; Chips	Never/ seldom	At least 1-3 times per month
Foods high in fat-3 points	Never/ seldom	At least 1-3 times per month

Cheese (high in saturated fat);<br>Butter (high in saturated fat);<br>Margarine (high content of trans fatty acids)		
Others-6 points<br>White bread (high glycemic index);<br>Pancakes /Belgian waffles; Cookies (high glycemic index, high trans fatty acids); Ice cream; Candy; Sugar	Never/ seldom	At least 1-3 times per month

Mediterranean Diet Score (MD Score) [81]

Component	Minimum score (0)	Maximum score (1)
Vegetables (g/d)	< median (sex-specific)	≥ median (sex-specific)
Legumes (g/d)	< median (sex-specific)	≥ median (sex-specific)
Fruits (g/d)	< median (sex-specific)	≥ median (sex-specific)
Cereals (g/d)	< median (sex-specific)	≥ median (sex-specific)
Fish (g/d) *	< median (sex-specific)	≥ median (sex-specific)
Red meat (g/d)	≥ median (sex-specific)	< median (sex-specific)
Dairy products (g/d)	≥ median (sex-specific)	< median (sex-specific)
Alcohol (g/d)	Men: <10 or >50 g/d<br>Women: <5 or >25g/d	Men: 10-50 g/d<br>Women: 5-25 g/d
Olive oil (g/d) **	< median (sex-specific)	≥ median (sex-specific)

Binary scoring method; * Fish was added and **olive oil was replaced monounsaturated fat (MUFA): saturated fat (SFA) in the reviewed studies.

Mediterranean Diet Pattern Index (MDP Index) [82]

Component	Minimum score (0)	Maximum score (1)
Vegetables (g/d)	≤ median (sex-specific)	> median (sex-specific)
Legumes (g/d)	≤ median (sex-specific)	> median (sex-specific)
Fruits and nuts (g/d)	≤ median (sex-specific)	> median (sex-specific)
Cereals (g/d)	≤ median (sex-specific)	> median (sex-specific)
Fish (g/d)	≤ median (sex-specific)	> median (sex-specific)
Meat and meat products (g/d)	> median (sex-specific)	≤ median (sex-specific)
Dairy products (g/d)	> median (sex-specific)	≤ median (sex-specific)
Alcohol (g/d)	<5 or >25 g/d	5-25 g/d
Monounsaturated fat (MUFA): Saturated fat (SFA)	≤ median (sex-specific)	> median (sex-specific)

Binary scoring method;

MedDiet Score [83]

Component	Minimum score (0)	Maximum score (1)
Cereals (g/d)	< median (sex-specific)	≥ median (sex-specific)
Vegetables (g/d)	< median (sex-specific)	≥ median (sex-specific)
Legumes (g/d)	< median (sex-specific)	≥ median (sex-specific)
Fruits and Nuts (g/d)	< median (sex-specific)	≥ median (sex-specific)
Fish (g/d)	< median (sex-specific)	≥ median (sex-specific)
Dairy products (g/d)	≥ median (sex-specific)	< median (sex-specific)
Poultry (g/d)	≥ median (sex-specific)	< median (sex-specific)
Red meat (steak) (g/d)	≥ median (sex-specific)	< median (sex-specific)
Alcohol (g/d)**	Men: <10 or >50 g/d<br>Women: <5 or >25g/d	Men: 10-50 g/d<br>Women: 5-25 g/d
Monounsaturated fat (MUFA): saturated fat (SFA)*	< median	≥ median

Omega-6: Omega-3*	< median	≥ median
Egg*	< median or > median	0-4 eggs/week

Binary scoring method; **Alcohol was not included in the reviewed study; *MUFA: SFA, omega-6: omega-3 and egg were added in the reviewed study (olive oil in the original versions).

Mediterranean Diet Scale (MDS) [84]

Component	Minimum score (0)	Maximum score (1)
Vegetables (excluding potatoes)	< median (sex-specific)	≥ median (sex-specific)
Legumes	< median (sex-specific)	≥ median (sex-specific)
Fruits and Nuts	< median (sex-specific)	≥ median (sex-specific)
Cereals	< median (sex-specific)	≥ median (sex-specific)
Fish and seafood	< median (sex-specific)	≥ median (sex-specific)
Dairy products	≥ median (sex-specific)	< median (sex-specific)
Meat and meat products	≥ median (sex-specific)	< median (sex-specific)
Alcohol*	Men: <10 or >50 g/d<br>Women: <5 or >25g/d	Men: 10-50 g/d<br>Women: 5-25 g/d
Lipid ratios**	< median	≥ median

Binary scoring method; * Alcohol intake was modified in applied Australian studies as 0 or > 2 times per day (scored 0) and ≤2 times per day (scored 1).** Lipid ratios were not included in the reviewed studies.

Dietary Approach to Stop Hypertension (DASH) [85]

Component	Foods	Scoring criteria
Fruits	All fruits and fruit juices (servings/d)	Q1= 1 point<br>Q2= 2 points<br>Q3= 3 points<br>Q4= 4 points<br>Q5= 5 points
Vegetables	All vegetables except potatoes and legumes (servings/d)	
Nuts and legumes	Nuts and peanut butter, dried beans, peas, tofu (servings/d)	
Whole grains	Brown rice, dark breads, cooked cereal, whole grain cereal, other grains, popcorn, wheat germ, bran (servings/d)	
Low-fat dairy	Skim milk, yogurt, cottage cheese (servings/d)	
Sodium	Sum of sodium content of all foods in FFQ (mg)	Reverse scoring<br>Q1= 5 point<br>Q2= 4 points<br>Q3= 3 points<br>Q4= 2 points<br>Q5= 1points
Red and processed meats	Beef, pork, lamb, deli meats, organ meats, hot dogs, bacon (servings/d)	
Sweetened beverages	Carbonated and noncarbonated sweetened beverages (servings/d)	

Alternative Healthy Eating Index (AHEI) [86]

Component	Criteria for minimum score of 0**	Criteria for maximum score of 10**
Vegetables (servings/d)	0	5
Fruits (servings/d)	0	4
Nuts and soy protein (servings/d)	0	1
Ratio of white to red meat	0	4
Cereal fibre (g/d)	0	15
Trans-fat (% of energy)	≥4	≤0.5
Polyunsaturated fatty acid: Saturated fatty acid (P: S)	≤0.1	≥1
Alcohol (beer, wine, liquor) (servings/d)	Men: 0 or >3.5<br>Women: 0 or >2.5	Men: 1.5-2.5<br>Women: 0.5-1.5
Multivitamin use*	Non-use or < 5 years	Used ≥ 5 years
Total score	2.5	87.5

*Minimum score for multivitamin use=2.5, maximum score for multivitamin use=7.5; ** Intermediate intakes were scored proportionally except multivitamin use.

Alternative Healthy Eating Index-2010 (AHEI-2010) [87]

Component	Criteria for minimum score of 0*	Criteria for maximum score of 10*
Vegetables, except potatoes (servings/d)	0	≥5
Whole fruits (servings/d)	0	≥4
Whole grains (g/d)<br>Women<br>Men	 0<br>0	 75<br>90
Sugar-sweetened beverages and fruit juice (servings/d)	≥1	0
Nuts and legumes (servings/d)	0	≥1
Red/ processed meat (servings/d)	≥1.5	0
Trans-fat (% of energy)	≥4	≤0.5
Long-chain (n-3) fats (EPA+DHA), mg/d	0	250
PUFA (% of energy)	≤2	≥10
Sodium (mg/d)	Highest decile	Lowest decile
Alcohol (drinks/d)<br>Women<br>Men	 ≥2.5<br>≥3.5	 0.5-1.5<br>0.5-2.0

*Intermediate amount scored proportionately

Diet Quality Tool (DQT) [57]

Food items*	Minimum score (0)	Maximum score (10)
Serves of vegetables eaten/day	I don't eat vegetables	≥6 serves
Serves of fruit eaten/day	I don't eat fruit	≥6 serves
Type of rice, pasta or noodles	I do not eat rice, pasta or noodles	Brown/ wholemeal/ wholegrain
Type of breakfast cereal (muesili, or wholegrain cereal such as Wheet-bix, Vitabrit, Wheeties, porridge, All Bran, Branflakes, Sultana Bran, Fibreplus)	No/I don't eat breakfast cereal	Yes
Type of bread	I don't eat bread	Wholegrain or multi-grain bread
Type of spread	I don't use any fat spread	Margarine of any kind
Type of milk	None	Soya milk
Trimming meat fat before or after cooking	I don't eat meat	Usually
Eating pies, pastries, fried foods, hot chips or takeaway meals	Pieces taken per day	I don't eat those foods
Eating biscuits, cakes, chocolate, lollies, ice cream or crips	Pieces taken per day	I don't eat those foods
Type of fish	I do not eat fish	Oily (salmon, sardine, tuna, mackerel, trevally)
Adding salt to food after cooking	Usually	Never/rarely
Adding salt to food during cooking	Usually	Never/rarely

*Intakes between minimum and maximum scored proportionately

Dietary Inflammatory Index (DII) [88]

Components	
Nutrients<br>Carbohydrate; protein; total fat; saturated, monosaturated, and polyunsaturated fats; omega-3 and omega FAs; cholesterol; vitamins A. B-6, B-12, C, D, and E; thiamin; riboflavin; niacin; iron; magnesium; zinc; selenium; folate; β-carotene; isoflavones; trans fat; flavonols; flavonones; anthocyanins	Dietary data were first linked to world database which provided a mean and standard deviation for each food parameter, that later became the multipliers. Then standard global mean as z score was calculated; by subtracting the standard global mean from the reported amount and dividing this by the global standard deviation of the world population (from 11 data sets). The resultant z scores were converted to proportions and centered by doubling and subtracting 1. A food parameter-specific DII score was obtained by multiplication of the centered percentile score with the respective inflammatory effect score; the summation of these individual DII scores generate the overall DII score.
Spices<br>Eugenol; garlic; ginger; onion; turmeric; falan-3-ol; falvones; pepper; thyme; oregano; rosemary	
Whole food and other<br>Alcohol; caffeine; green tea; black tea; fibre;	

*Based on the effect of the food parameter on inflammation; '+1' was assigned if pro-inflammatory (increased IL-1β, IL-6, TNF-α or CRP, or decreased IL-4 or IL-10); '-1' if anti-inflammatory (decreased IL-1β, IL-6, TNF-α or CRP, or increased IL-4 or IL-10); and '0' if the food parameter did not produce any changes in the inflammatory marker

References

1. Alhazmi A, Stojanovski E, McEvoy M, Brown W, Garg ML: **Diet quality score is a predictor of type 2 diabetes risk in women: The Australian Longitudinal Study on Women's Health.** *Br J Nutr* 2014, **112:**945-951.
2. Aljadani HMA, Sibbritt D, Patterson A, Clare C: **The Australian Recommended Food Score did not predict weight gain in middle-aged Australian women during six years of follow-up.** *Aust N Z J Public Health* 2013, **37:**322-328.
3. Aljadani HA, Patterson A, Sibbritt D, Hutchesson MJ, Jensen ME, Collins CE: **Diet quality measured by fruit and vegetable intake predicts weight change in young women.** *J Obes* 2013, **2013:**525161.
4. Aljadani HM, Patterson AJ, Sibbritt D, Collins CE: **Diet quality and 6-year risk of overweight and obesity among mid-age Australian women who were initially in the healthy weight range.** *Health Promot J Austr* 2016, **27:**29-35.
5. Arabshahi S, Lahmann PH, Williams GM, Marks GC, van der Pols JC: **Longitudinal change in diet quality in Australian adults varies by demographic, socio-economic, and lifestyle characteristics.** *J Nutr* 2011, **141:**1871-1879.
6. Arabshahi S, van der Pols JC, Williams GM, Marks GC, Lahmann PH: **Diet quality and change in anthropometric measures: 15-year longitudinal study in Australian adults.** *Br J Nutr* 2012, **107:**1376-1385.
7. Ashton LM, Morgan PJ, Hutchesson MJ, Rollo ME, Collins CE: **Feasibility and preliminary efficacy of the 'HEYMAN'healthy lifestyle program for young men: a pilot randomised controlled trial.** *Nutr J* 2017, **16:**2.
8. Ashton L, Williams R, Wood L, Schumacher T, Burrows T, Rollo M, Pezdirc K, Callister R, Collins C: **Comparison of Australian Recommended Food Score (ARFS) and plasma carotenoid concentrations: a validation study in adults.** *Nutrients* 2017, **9:**888.
9. Ashton L, Pezdirc K, Hutchesson M, Rollo M, Collins C: **Is skin coloration measured by reflectance spectroscopy related to intake of nutrient-dense foods? A cross-sectional evaluation in Australian young adults.** *Nutrients* 2018, **10:**11.
10. Backholer K, Spencer E, Gearon E, Magliano DJ, McNaughton SA, Shaw JE, Peeters A: **The association between socio-economic position and diet quality in Australian adults.** *Public Health Nutr* 2016, **19:**477-485.

11. Baker AL, Turner A, Kelly PJ, Spring B, Callister R, Collins CE, Woodcock KL, Kay-Lambkin FJ, Devir H, Lewin TJ: **'Better Health Choices' by telephone: a feasibility trial of improving diet and physical activity in people diagnosed with psychotic disorders.** *Psychiatry Res* 2014, **220:**63-70.
12. Bivoltsis A, Trapp G, Knuiman M, Hooper P, Ambrosini G: **Can a simple dietary index derived from a sub-set of questionnaire items assess diet quality in a sample of Australian adults?** *Nutrients* 2018, **10:**486.
13. Collins CE, Young AF, Hodge A: **Diet quality is associated with higher nutrient intake and self-rated health in mid-aged women.** *J Am Coll Nutr* 2008, **27:**146-157.
14. Collins CE, Patterson A, Fitzgerald D: **Higher diet quality does not predict lower Medicare costs but does predict number of claims in mid-aged Australian women.** *Nutrients* 2011, **3:**40-48.
15. Collins CE, Burrows TL, Rollo ME, Boggess MM, Watson JF, Guest M, Duncanson K, Pezdirc K, Hutchesson MJ: **The comparative validity and reproducibility of a diet quality index for adults: The Australian Recommended Food Score.** *Nutrients* 2015, **7:**785-798.
16. Crichton GE, Bryan J, Hodgson JM, Murphy KJ: **Mediterranean diet adherence and self-reported psychological functioning in an Australian sample.** *Appetite* 2013, **70:**53-59.
17. Davison B, Saeedi P, Black K, Harrex H, Haszard J, Meredith-Jones K, Quigg R, Skeaff S, Stoner L, Wong J: **The association between parent diet quality and child dietary patterns in nine-to eleven-year-old children from Dunedin, New Zealand.** *Nutrients* 2017, **9:**483.
18. Dugué PA, Hodge AM, Brinkman MT, Bassett JK, Shivappa N, Hebert JR, Hopper JL, English DR, Milne RL, Giles GG: **Association between selected dietary scores and the risk of urothelial cell carcinoma: A prospective cohort study.** *Int J Cancer* 2016, **139:**1251-1260.
19. Forsyth AK, Williams PG, Deane FP: **Nutrition status of primary care patients with depression and anxiety.** *Aust J Prim Health* 2012, **18:**172-176.
20. Forsyth A, Deane FP, Williams P: **A lifestyle intervention for primary care patients with depression and anxiety: a randomised controlled trial.** *Psychiatry Res* 2015, **230:**537-544.
21. Froud A, Murphy J, Cribb L, Ng CH, Sarris J: **The relationship between dietary quality, serum brain-derived neurotrophic factor (BDNF) level, and the Val66met polymorphism in predicting depression.** *Nutr Neurosci* 2019, **22:**513-521.
22. Gopinath B, Harris D, Flood V, Burlutsky G, Mitchell P: **A better diet quality is associated with a reduced likelihood of CKD in older adults.** *Nutr Metab Cardiovasc Dis* 2013, **23:**937-943.
23. Gopinath B, Flood VM, Wang JJ, Rochtchina E, Wong TY, Mitchell P: **Is quality of diet associated with the microvasculature? An analysis of diet quality and retinal vascular calibre in older adults.** *Br J Nutr* 2013, **110:**739-746.
24. Gopinath B, Rochtchina E, Flood V, Mitchell P: **Diet quality is prospectively associated with incident impaired fasting glucose in older adults.** *Diabet Med* 2013, **30:**557-562.
25. Gopinath B, Schneider J, Flood V, McMahon C, Burlutsky G, Leeder S, Mitchell P: **Association between diet quality with concurrent vision and hearing impairment in older adults.** *J Nutr Health Aging* 2014, **18:**251-256.
26. Gopinath B, Russell J, Flood VM, Burlutsky G, Mitchell P: **Adherence to dietary guidelines positively affects quality of life and functional status of older adults.** *J Acad Nutr Diet* 2014, **114:**220-229.

27. Gopinath B, Russell J, Kifley A, Flood VM, Mitchell P: **Adherence to dietary guidelines and successful aging over 10 years.** *J Gerontol A Biol Sci Med Sci* 2016, **71:**349-355.
28. Grech A, Sui Z, Siu HY, Zheng M, Allman-Farinelli M, Rangan A: **Socio-demographic determinants of diet quality in Australian adults using the validated Healthy Eating Index for Australian Adults (HEIFA-2013).** *Health Care (Don Mills)* 2017, **5:**7.
29. Grech A, Rangan A, Allman-Farinelli M: **Social determinants and poor diet quality of energy-dense diets of Australian young adults.** *Health Care (Don Mills)* 2017, **5:**70.
30. Harbury C, Collins CE, Callister R: **Diet quality is lower among adults with a BMI ≥40 kg m^{-2} or a history of weight loss surgery.** *Obes Res Clin Pract* 2019, **13:**197-204.
31. Hendrie GA, Rebuli MA, Golley RK: **Reliability and relative validity of a diet index score for adults derived from a self-reported short food survey.** *Nutr Diet* 2017, **74:**291-297.
32. Hendrie GA, Baird D, Golley RK, Noakes M: **The CSIRO Healthy Diet Score: An online survey to estimate compliance with the Australian Dietary Guidelines.** *Nutrients* 2017, **9:**47.
33. Hendrie G, Golley R, Noakes M: **Compliance with dietary guidelines varies by weight status: A cross-sectional study of Australian adults.** *Nutrients* 2018, **10:**197.
34. Hodge A, Bassett J, Shivappa N, Hébert J, English D, Giles G, Severi G: **Dietary inflammatory index, Mediterranean diet score, and lung cancer: a prospective study.** *Cancer Causes Control* 2016, **27:**907-917.
35. Hodge AM, Bassett JK, Dugué P-A, Shivappa N, Hébert JR, Milne R, English DR, Giles GG: **Dietary inflammatory index or Mediterranean diet score as risk factors for total and cardiovascular mortality.** *Nutr Metab Cardiovasc Dis* 2018, **28:**461-469.
36. Hong T, Flood V, Rochtchina E, Mitchell P, Russell J, Wang JJ: **Adherence to dietary guidelines and the 10-year cumulative incidence of visual impairment: the Blue Mountains Eye Study.** *Am J Ophthalmol* 2014, **158:**302-308.
37. Kullen CJ, Farrugia J-L, Prvan T, O'Connor HT: **Relationship between general nutrition knowledge and diet quality in Australian military personnel.** *Br J Nutr* 2016, **115:**1489-1497.
38. Lai JS, Oldmeadow C, Hure AJ, McEvoy M, Byles J, Attia J: **Longitudinal diet quality is not associated with depressive symptoms in a cohort of middle-aged Australian women.** *Br J Nutr* 2016, **115:**842-850.
39. Lai JS, Hure AJ, Oldmeadow C, McEvoy M, Byles J, Attia J: **Prospective study on the association between diet quality and depression in mid-aged women over 9 years.** *Eur J Nutr* 2017, **56:**273-281.
40. Leech RM, Livingstone KM, Worsley A, Timperio A, McNaughton SA: **Meal frequency but not snack frequency is associated with micronutrient intakes and overall diet quality in Australian men and women.** *J Nutr* 2016, **146:**2027-2034.
41. Leech RM, Timperio A, Livingstone KM, Worsley A, McNaughton SA: **Temporal eating patterns: associations with nutrient intakes, diet quality, and measures of adiposity.** *Am J Clin Nutr* 2017, **106:**1121-1130.
42. Livingstone KM, McNaughton SA: **Diet quality is associated with obesity and hypertension in Australian adults: a cross sectional study.** *BMC Public Health* 2016, **16:**1037.

43. Livingstone K, Olstad D, Leech R, Ball K, Meertens B, Potter J, Cleanthous X, Reynolds R, McNaughton S: **Socioeconomic inequities in diet quality and nutrient intakes among Australian adults: findings from a nationally representative cross-sectional study.** *Nutrients* 2017, **9:**1092.
44. Livingstone KM, McNaughton SA: **Association between diet quality, dietary patterns and cardiometabolic health in Australian adults: a cross-sectional study.** *Nutr J* 2018, **17:**19.
45. Martin J, Moran L, Teede H, Ranasinha S, Lombard C, Harrison C: **Exploring diet quality between urban and rural dwelling women of reproductive age.** *Nutrients* 2017, **9:**586.
46. Martin J, Moran L, Teede H, Ranasinha S, Lombard C, Harrison C: **Diet quality in a weight gain prevention trial of reproductive aged women: a secondary analysis of a cluster randomized controlled trial.** *Nutrients* 2019, **11:**49.
47. Mayr HL, Itsiopoulos C, Tierney AC, Ruiz-Canela M, Hebert JR, Shivappa N, Thomas CJ: **Improvement in dietary inflammatory index score after 6-month dietary intervention is associated with reduction in interleukin-6 in patients with coronary heart disease: The AUSMED heart trial.** *Nutr Res* 2018, **55:**108-121.
48. McLeod ER, Campbell KJ, Hesketh KD: **Nutrition knowledge: a mediator between socioeconomic position and diet quality in Australian first-time mothers.** *J Am Diet Assoc* 2011, **111:**696-704.
49. McNaughton SA, Ball K, Crawford D, Mishra GD: **An index of diet and eating patterns is a valid measure of diet quality in an Australian population.** *J Nutr* 2008, **138:**86-93.
50. McNaughton SA, Dunstan DW, Ball K, Shaw J, Crawford D: **Dietary quality Is associated with diabetes and cardio-metabolic risk factors.** *J Nutr* 2009, **139:**734-742.
51. Milte CM, Thorpe MG, Crawford D, Ball K, McNaughton SA: **Associations of diet quality with health-related quality of life in older Australian men and women.** *Exp Gerontol* 2015, **64:**8-16.
52. Milte CM, Russell AP, Ball K, Crawford D, Salmon J, McNaughton SA: **Diet quality and telomere length in older Australian men and women.** *Eur J Nutr* 2018, **57:**363-372.
53. Morrison M, Koh D, Lowe J, Miller YD, Marshall AL, Colyvas K, Collins C: **Postpartum diet quality in Australian women following a gestational diabetes pregnancy.** *Eur J Clin Nutr* 2012, **66:**1160-1165.
54. Nagle C, Ibiebele T, Shivappa N, Hébert J, DeFazio A, Webb P, Study AOC: **The association between the inflammatory potential of diet and risk of developing, and survival following, a diagnosis of ovarian cancer.** *Eur J Nutr* 2019, **58:**1747-1756.
55. O'Brien KM, Hutchesson MJ, Jensen M, Morgan P, Callister R, Collins CE: **Participants in an online weight loss program can improve diet quality during weight loss: a randomized controlled trial.** *Nutr J* 2014, **13:**82.
56. Olstad DL, Lamb KE, Thornton LE, McNaughton SA, Crawford DA, Minaker LM, Ball K: **Prospective associations between diet quality and body mass index in disadvantaged women: the Resilience for Eating and Activity Despite Inequality (READI) study.** *Int J Epidemiol* 2017, **46:**1433-1443.
57. O'Reilly S, McCann L: **Development and validation of the Diet Quality Tool for use in cardiovascular disease prevention settings.** *Aust J Prim Health* 2012, **18:**138-147.
58. Petersen KS, Blanch N, Wepener RH, Clifton PM, Keogh JB: **Dietary quality in people with type 1 and type 2 diabetes compared to age, sex and BMI matched controls.** *Diabetes Res Clin Pract* 2015, **107:**e7-e10.

59. Potter J, Collins C, Brown L, Hure A: **Diet quality of Australian breast cancer survivors: a cross-sectional analysis from the Australian Longitudinal Study on Women's Health.** *J Hum Nutr Diet* 2014, **27:**569-576.
60. Reeves MM, Healy GN, Owen N, Shaw JE, Zimmet PZ, Dunstan DW: **Joint associations of poor diet quality and prolonged television viewing time with abnormal glucose metabolism in Australian men and women.** *Prev Med* 2013, **57:**471-476.
61. Ribeiro RV, Hirani V, Senior AM, Gosby AK, Cumming RG, Blyth FM, Naganathan V, Waite LM, Handelsman DJ, Kendig H: **Diet quality and its implications on the cardio-metabolic, physical and general health of older men: the Concord Health and Ageing in Men Project (CHAMP).** *Br J Nutr* 2017, **118:**130-143.
62. Roach LA, Lambert K, Holt JL, Meyer BJ: **Diet quality in patients with end-stage kidney disease undergoing dialysis.** *J Ren Care* 2017, **43:**226-234.
63. Roy R, Hebden L, Rangan A, Allman-Farinelli M: **The development, application, and validation of a Healthy Eating Index for Australian Adults (HEIFA-2013).** *Nutrition* 2016, **32:**432-440.
64. Roy R, Rangan A, Hebden L, Louie JCY, Tang LM, Kay J, Allman-Farinelli M: **Dietary contribution of foods and beverages sold within a university campus and its effect on diet quality of young adults.** *Nutrition* 2017, **34:**118-123.
65. Russell J, Flood V, Rochtchina E, Gopinath B, Allman-Farinelli M, Bauman A, Mitchell P: **Adherence to dietary guidelines and 15-year risk of all-cause mortality.** *Br J Nutr* 2013, **109:**547–555.
66. Russell JC, Flood VM, Sadeghpour A, Gopinath B, Mitchell P: **Total Diet Score as a valid method of measuring diet quality among older adults.** *Asia Pac J Clin Nutr* 2017, **26:**212-219.
67. Shivappa N, Schoenaker DAJM, Hebert JR, Mishra GD: **Association between inflammatory potential of diet and risk of depression in middle-aged women: the Australian Longitudinal Study on Women's Health.** *Br J Nutr* 2016, **116:**1077-1086.
68. Smith KJ, McNaughton SA, Gall SL, Otahal P, Dwyer T, Venn AJ: **Associations between partnering and parenting transitions and dietary habits in young adults.** *J Acad Nutr Diet* 2017, **117:**1210-1221.
69. Thorpe MG, Kestin M, Riddell LJ, Keast RS, McNaughton SA: **Diet quality in young adults and its association with food-related behaviours.** *Public Health Nutr* 2013, **17:**1767-1775.
70. Thorpe MG, Milte CM, Crawford D, McNaughton SA: **A revised Australian Dietary Guideline Index and its association with key sociodemographic factors, health behaviors and body mass Index in peri-retirement aged adults.** *Nutrients* 2016, **8:**160.
71. Vissers LET, Waller MA, van der Schouw YT, Hebert JR, Shivappa N, Schoenaker DAJM, Mishra GD: **The relationship between the dietary inflammatory index and risk of total cardiovascular disease, ischemic heart disease and cerebrovascular disease: Findings from an Australian population-based prospective cohort study of women.** *Atherosclerosis* 2016, **253:**164-170.
72. Vissers LET, Waller M, van der Schouw YT, Hébert JR, Shivappa N, Schoenaker DAJM, Mishra GD: **A pro-inflammatory diet is associated with increased risk of developing hypertension among middle-aged women.** *Nutr Metab Cardiovasc Dis* 2017, **27:**564-570.
73. Williams RL, Rollo ME, Schumacher T, Collins CE: **Diet quality scores of Australian adults who have completed the Healthy Eating Quiz.** *Nutrients* 2017, **9:**880.

74. Wong JE, Haszard JJ, Howe AS, Parnell WR, Skidmore PML: **Development of a Healthy Dietary Habits Index for New Zealand adults.** *Nutrients* 2017, **9:**454-465.
75. Wood LG, Shivappa N, Berthon BS, Gibson PG, Hebert JR: **Dietary inflammatory index is related to asthma risk, lung function and systemic inflammation in asthma.** *Clin Exp Allergy* 2015, **45:**177-183.
76. Zarrin R, Ibiebele TI, Marks GC: **Development and validity assessment of a diet quality index for Australians.** *Asia Pac J Clin Nutr* 2013, **22:**177-187.
77. Australian Institute of Health and Welfare: *Australian diet quality index project.* Canberra: Australian Institute of Health and Welfare; 2007.
78. Haines PS, Siega-Riz AM, Popkin BM: **The Diet Quality Index Revised: A measurement instrument for populations** *J Am Diet Assoc* 1999, **99:**697–704.
79. Kant AK, Schatzkin A, Graubard BI, Schairer C: **A prospective study of diet quality and mortality in women.** *JAMA* 2000, **283:**2109-2115.
80. Michels KB, Wolk A: **A prospective study of variety of healthy foods and mortality in women.** *Int J Epidemiol* 2002, **31:**847-854.
81. Trichopoulou A, Kouris-Blazos A, Wahlqvist ML, Gnardellis C, Lagiou P, Polychronopoulos E, Vassilakou T, Lipworth L, Trichopoulos D: **Diet and overall survival in elderly people.** *BMJ* 1995, **311:**1457-1460.
82. Trichopoulou A, Costacou T, Bamia C, Trichopoulos D: **Adherence to a Mediterranean Diet and Survival in a Greek Population.** *N Engl J Med* 2003, **348:**2599-2608.
83. Trichopoulou A, Orfanos P, Norat T, Bueno-de-Mesquita B, Ocké M, Peeters PH, van der Schouw YT, Boeing H, Hoffmann K, Boffetta P, et al: **Modified Mediterranean diet and survival: EPIC-elderly prospective cohort study.** *BMJ* 2005, **330:**991-998.
84. Hodge A, English D, Itsiopoulos C, O'dea K, Giles G: **Does a Mediterranean diet reduce the mortality risk associated with diabetes: evidence from the Melbourne Collaborative Cohort Study.** *Nutr Metab Cardiovasc Dis* 2011, **21:**733-739.
85. Fung TT, Chiuve SE, McCullough ML, Rexrode KM, Logroscino G, Hu FB: **Adherence to a DASH-style diet and risk of coronary heart disease and stroke in women.** *Arch Intern Med* 2008, **168:**713-720.
86. McCullough ML, Feskanich D, Stampfer MJ, Giovannucci EL, Rimm EB, Hu FB, Spiegelman D, Hunter DJ, Colditz GA, Willett WC: **Diet quality and major chronic disease risk in men and women: moving toward improved dietary guidance.** *Am J Clin Nutr* 2002, **76:**1261-1271.
87. Chiuve SE, Fung TT, Rimm EB, Hu FB, McCullough ML, Wang M, Stampfer MJ, Willett WC: **Alternative dietary indices both strongly predict risk of chronic disease.** *J Nutr* 2012, **142:**1009-1018.
88. Shivappa N, Steck SE, Hurley TG, Hussey JR, Hébert JR: **Designing and developing a literature-derived, population-based dietary inflammatory index.** *Public Health Nutr* 2014, **17:**1689-1696.
89. Popkin BM, Armstrong LE, Bray GM, Caballero B, Frei B, Willett WC: **A new proposed guidance system for beverage consumption in the United States.** *The American journal of clinical nutrition* 2006, **83:**529-542.
